# Using photocaging for fast time-resolved structural biology studies

**DOI:** 10.1107/S2059798321008809

**Published:** 2021-09-22

**Authors:** Diana C. F. Monteiro, Emmanuel Amoah, Cromarte Rogers, Arwen R. Pearson

**Affiliations:** a Hauptman–Woodward Medical Research Institute, 700 Ellicot Street, Buffalo, NY 14203, USA; b The Hamburg Centre for Ultrafast Imaging, Universität Hamburg, Luruper Chaussee 149, 22761 Hamburg, Germany; c Department of Chemistry, Universität Hamburg, Martin-Luther-King-Platz 6, 20146 Hamburg, Germany; d Department of Physics, Universität Hamburg, Luruper Chaussee 149, 22761 Hamburg, Germany

**Keywords:** time-resolved structural biology, photocages, reaction initiation, serial crystallography

## Abstract

Careful selection of photocaging approaches is critical to achieve fast and well synchronized reaction initiation and perform successful time-resolved structural biology experiments. This review summarizes the best characterized and most relevant photocaging groups previously described in the literature. It also provides a walkthrough of the essential factors to consider in designing a suitable photocaged molecule to address specific biological questions, focusing on photocaging groups with well characterized spectroscopic properties. The relationships between decay rates (*k* in s^−1^), quantum yields (φ) and molar extinction coefficients (ɛ_max_ in *M* ^−1^ cm^−1^) are highlighted for different groups. The effects of the nature of the photocaged group on these properties is also discussed. Four main photocaging scaffolds are presented in detail, *o*-nitrobenzyls, *p*-hydroxyphenyls, coumarinyls and nitrodibenzofuranyls, along with three examples of the use of this technology. Furthermore, a subset of specialty photocages are highlighted: photoacids, molecular photoswitches and metal-containing photocages. These extend the range of photocaging approaches by, for example, controlling pH or generating conformationally locked molecules.

## Abstract 2

This review summarizes the best characterized and most relevant photocaging groups for time-resolved structural biology described in the literature to date. It provides a walkthrough of the essential factors to consider in designing a suitable photocaged molecule to address specific biological questions using time-resolved X-ray diffraction or solution-scattering methods.

## 1. Introduction   

**Figure 1:**
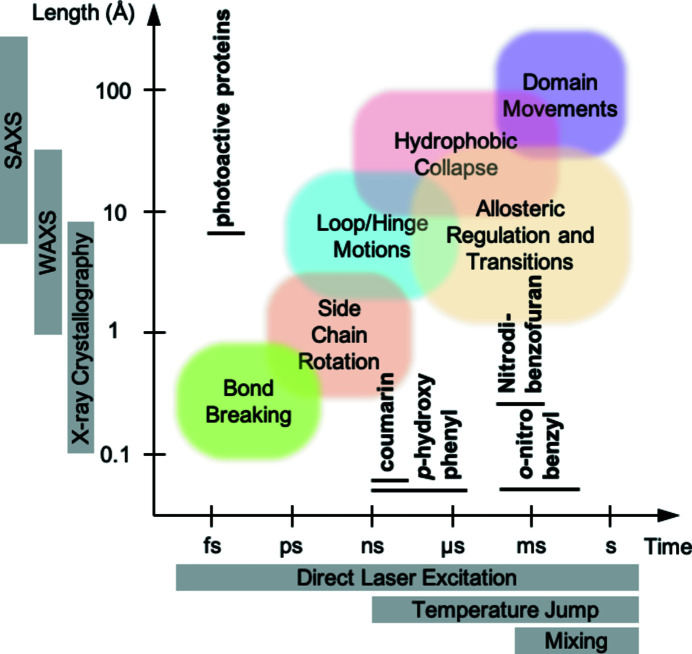
Achievable time resolutions for different protein-activation methods (Levantino, Yorke *et al.*, 2015 ▸). Different protein transitions are depicted along with their typical timescales and length scales. X-ray crystallo­graphy, small-angle X-ray scattering (SAXS) and wide-angle X-ray scattering (WAXS) can capture different types of structural transitions. Direct laser excitation, temperature jumps and mixing are the usual approaches to sample triggering and synchronization. The typical decaging half-lives of the four main photocaging groups addressed in this review (coumarin, *p*-hydroxyphenyl, *o*-nitrobenzyl and nitrodi­benzofuran) are shown as black bars.

The timescales of interest in biomolecular science span a wide range, from local reaction chemistry occurring on femto­second (10^−15^ s) to nanosecond (10^−9^ s) timescales to long-range motions (changes in macromolecular conformation) occurring over much slower timescales (tens of milliseconds to seconds; Fig. 1 ▸). These small- and large-scale motions often gate the reaction chemistry and link to biological responses such as signaling or complex assembly. To understand biological processes fully at the molecular level, we require the ability to ‘watch’ the molecules as they react or transform in *real time*, structurally determining the transient species and intermediates that occur, which are often short-lived.

Time-resolved structural biology has been possible for decades. The use of pump–probe Laue crystallography to achieve submillisecond time resolutions was first demonstrated in the 1990s (Moffat, 2019 ▸). In such experiments, the reaction of interest is triggered in an ensemble of biomacromolecules (usually with light), and the structure is probed after a pre-defined time delay using an X-ray pulse. Different time delays yield a dynamically resolved stop-motion-like visualization of the protein during activity. The achievable time resolution of the experiment is determined by whichever is the slowest: the time required for reaction initiation or the length of the probing X-ray pulse (Helliwell & Rentzepis, 1997 ▸; Moffat, 1998 ▸, 2001 ▸).

The emergence of extremely bright X-ray sources, such as third- and fourth-generation synchrotrons and X-ray free-electron lasers (XFELs), as well as advances in hardware such as improved X-ray area detectors (which can now reach readout rates of hundreds of hertz), have made a huge range of time resolutions accessible for X-ray diffraction and scattering methods (Šrajer & Schmidt, 2017 ▸; Levantino, Yorke *et al.*, 2015 ▸; Neutze & Moffat, 2012 ▸), ranging from femtoseconds for XFELs (Behrens *et al.*, 2014 ▸; Neutze, 2014 ▸; Fromme, 2015 ▸; Chapman *et al.*, 2011 ▸) through hundreds of picoseconds for Laue radiation (Schotte *et al.*, 2003 ▸, 2012 ▸; Hajdu *et al.*, 1987 ▸) to milliseconds at monochromatic synchrotron sources (Schulz *et al.*, 2018 ▸; Beyerlein *et al.*, 2017 ▸; Mehrabi *et al.*, 2019 ▸; Martin-Garcia *et al.*, 2017 ▸). The rapid development of novel sample-delivery methods (Cheng, 2020 ▸; Grünbein & Nass Kovacs, 2019 ▸) has accompanied these advances, providing platforms for the fast sample refreshment which is needed for serial crystallography experiments. Such platforms utilize clever setups such as X-ray-compatible fixed targets (Schulz *et al.*, 2018 ▸; Roedig *et al.*, 2016 ▸) and enclosed microfluidics (Tosha *et al.*, 2017 ▸; Sui & Perry, 2017 ▸; Monteiro *et al.*, 2019 ▸, 2020 ▸) as well as liquid jets (Martiel *et al.*, 2019 ▸) and viscous jets (Grünbein & Nass Kovacs, 2019 ▸; Martin-Garcia *et al.*, 2017 ▸). All of these technological developments have led to a boost in interest in time-resolved structural biology, and a rapid increase in the number of systems that could be studied over the last decade. Yet, despite these great advances in both X-ray sources and sample-delivery methods, the fundamental roadblock to time-resolved structural biology remains reaction initiation. The macromolecules in the crystal or solution samples have to be synchronized in order to obtain a clear picture of the structural changes, and therefore the reaction must be triggered uniformly through the sample on a timescale that is commensurate with the reaction steps of interest.

## 2. Reaction initiation   

Uniformly triggering an ensemble of molecules quickly and accurately can be achieved using a variety of methods (Fig. 1 ▸). By far the most widely utilized method of activation makes use of laser pulses in the ultraviolet and visible light range. A high-intensity, short laser pulse is capable of delivering time resolutions in the femtosecond to nanosecond range (Grünbein *et al.*, 2020 ▸). Unsurprisingly, most experiments using this approach have focused on the study of naturally photoactivated macromolecules. Examples include those containing chromophores which can undergo *cis*–*trans* isomerization [*i.e.* rhodopsins (Malmerberg *et al.*, 2015 ▸; Nogly *et al.*, 2018 ▸), photoactive yellow protein (Cho *et al.*, 2016 ▸; Schmidt, 2017 ▸), phytochromes (Heyes *et al.*, 2019 ▸; Claesson *et al.*, 2020 ▸) and green fluorescent protein (Coquelle *et al.*, 2018 ▸)], photon-induced conformational changes [*i.e.* photosynthetic reaction center (Deisenhofer & Michel, 1989 ▸; Baxter *et al.*, 2004 ▸) and photosystem II (Kupitz *et al.*, 2014 ▸; Wöhri *et al.*, 2010 ▸; Baxter *et al.*, 2004 ▸)] or contain a bond that can be directly photolyzed (*i.e.* the cleavage of CO from myoglobin or hemoglobin; Levantino, Schirò *et al.*, 2015 ▸; Šrajer & Royer, 2008 ▸; Bourgeois *et al.*, 2006 ▸; Schotte *et al.*, 2012 ▸). These experiments have taken advantage of the fact that the systems under study are inherently light-activatable and that this activation is both extremely fast and very efficient. The speed of activation allows very high time resolutions to be achieved. The efficiency of activation translates into a large proportion of the protein molecules in the sample being activated, making the resulting structural changes easy to visualize.

Unfortunately (and rather unsurprisingly), less than 0.5% of proteins are naturally photoactivatable^1^ and so alternative approaches to reaction initiation must be found. One option is the use of infrared pulses to generate temperature jumps which overcome thermal activation barriers (Thompson *et al.*, 2019 ▸; Kubelka, 2009 ▸). Thermal activation is not as fast as direct photoactivation, but can deliver time resolutions of the order of nanoseconds. Alternatively, for slow reactions, where the desired time resolution is in the millisecond to second range, ligand diffusion through mixing is an ideal approach. Microfluidic rapid mixing experiments either in solution or using microcrystals (of at most 10–20 µm in their thickest dimension) are able to provide insight into millisecond dynamics (Schmidt, 2013 ▸; Makinen & Fink, 1977 ▸). Certain geometries employed with very small microcrystals (<1 µm) expand the time resolution to the submillisecond timescale (Calvey *et al.*, 2016 ▸). However, for cases where the crystals are grown in viscous media (for example LCP), rapid mixing cannot be employed as ligand diffusion is too slow. When the timescales of the reaction of interest are fast in comparison to the achievable diffusion speeds, light activation remains the only viable option. This requires methods capable of rendering proteins light-activatable and brings us to the concept of photocaging.

## 3. Photocaging principles   

Photocaging is a chemical approach that introduces a covalently bound photolabile protecting group (a photocage) onto a protein or its ligand, rendering the system inactive. Activation is achieved by a light pulse, which cleaves the photocage and releases the active molecule. Photocaging is not a one-size-fits-all approach and has to be tailored to the specific system and molecules under study. As with many other time-resolved setups, photo-decaging-based experiments present a multidimensional problem. Here, we provide a guide to the design of photocaged experiments and describe all of the necessary considerations.

Photocaging of bioactive molecules is a nontrivial process and, although the first studies date back to the 1970s (Kaplan *et al.*, 1978 ▸) and provide details regarding the synthesis, photolysis and use of these early compounds, information about the spectroscopic and chemical properties of many caged biomolecules is still scarce and incomplete. This lack of information is mainly due to the nature of the experiments for which many biocompatible photocages were initially developed. Most photocaged compounds have been used in cellular studies, where fast time resolution is not the main requirement. Instead, changes occurring on second or minute timescales are of interest and therefore the photocages are typically released using continuous, low-power, long-term illumination (Hagen, Benndorf *et al.*, 2005 ▸). In these cases, the rate of photocleavage is not determined and instead the long-term accumulation of the active compound is quoted. Some examples of such work include the study of slow-onset mechanisms such as calcium regulation by inositol (Hauke *et al.*, 2019 ▸) and cADP (Aarhus *et al.*, 1995 ▸), protein translocation (Pavlovic *et al.*, 2016 ▸) and microbial gene expression (Binder *et al.*, 2016 ▸) in living cells. To further complicate the transfer of this photocaging technology to fast time-resolved experiments, in the cases where the rates and yields of cleavage have been reported the results are often quoted without a reference to the power of the illumination source used. Furthermore, when comparing similar photocaging groups from different studies, the photolysis experiments are typically carried out using differing wavelengths. Given this scarcity of comparable information, the relative efficiency of different photocaging approaches can only be evaluated qualitatively.

**Figure 2:**
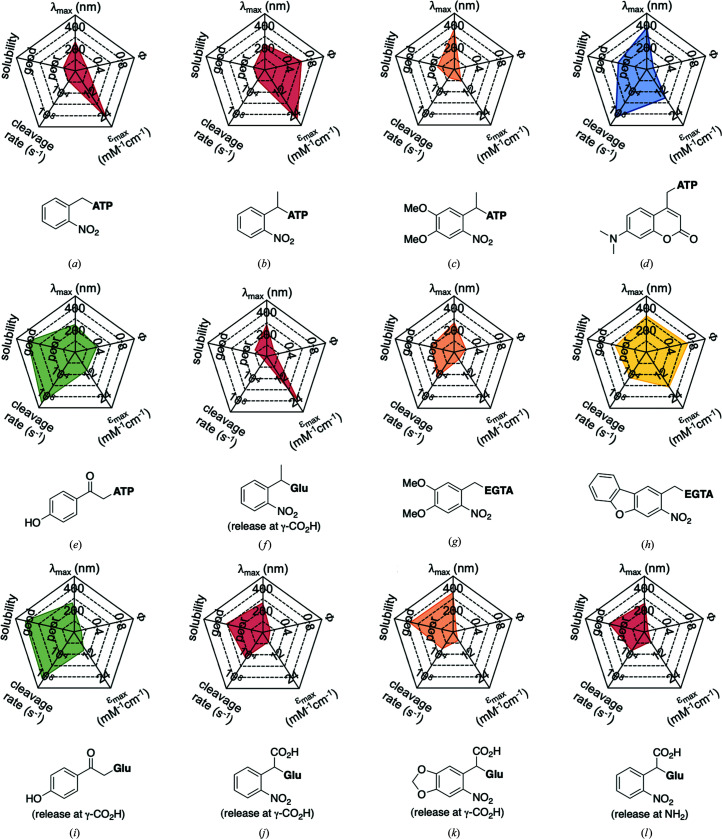
Examples of biologically relevant photocaged small molecules for which rates of photo-decaging have been reported. The compounds are ordered so as to facilitate comparison between similar scaffolds. Color-coding indicates compound classes: *o*-nitrobenzyl (red), red-shifted *o*-nitrobenzyl (orange), nitrodibenzylfuranyl (yellow), *p*-hydroxyphenyl (green) and coumarinyl (blue). Each plot gives values for five properties: maximum absorption wavelength (λ_max_), extinction coefficient at λ_max_ (ɛ_max_), cleavage rate, quantum yield (φ) and a qualitative assessment of the expected solubility. The photo-released groups are highlighted in **bold**. The point of photocage attachment for each compound is phosphate (ATP), γ-carboxylate or amine (Glu), or ether (EGTA), as shown in Fig. 3 ▸.

**Figure 3:**
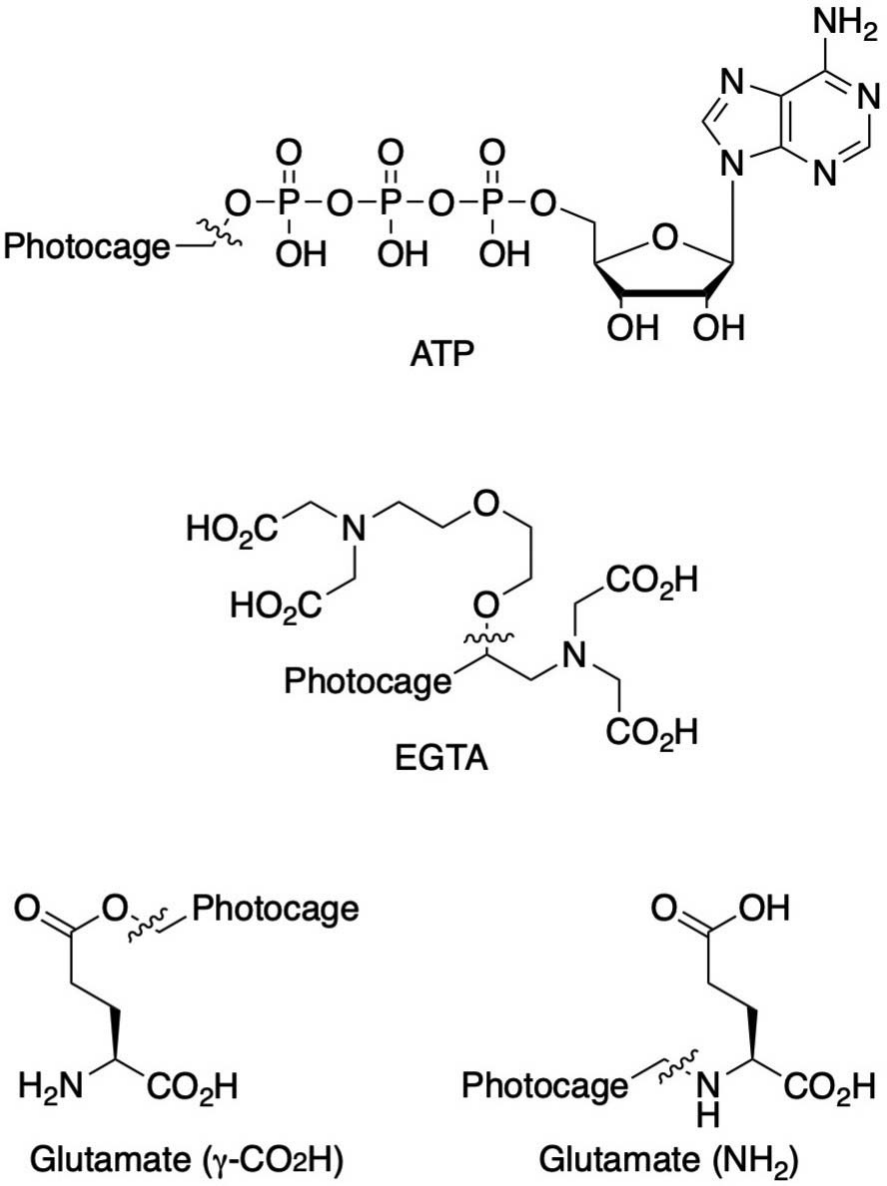
Full representation of the photocaged compounds shown in Fig. 2 ▸. The covalent bonds for attachment of the photocage to the small molecule and its release are highlighted by cleavage lines.

In the few cases where information on both the rate and the yield of photocleavage is available, the values are usually calculated from the fragmentation of the compounds following a short (nanosecond) high-power pulse of light. Some studies into the effects of substituents, leaving groups and cleavage conditions on the efficiency of photolysis and product release of photocages have been reported (Corrie *et al.*, 2005 ▸; Klán *et al.*, 2013 ▸). As this review focuses on the use of photocages for fast time-resolved structural biology experiments, the following discussion will focus on compounds for which rates of cleavage have been determined. Fig. 2 ▸ shows a comparison of most of the biologically relevant photocaged compounds reported to date for which photorelease rates have been reported. These compounds will be the basis of the main discussion in this review and can be grouped into four main scaffolds: *ortho*-nitrobenzyls (*o*NB; red/orange), coumarins (Cm; blue), *para*-hydroxyphenyls (*p*HP; green) and nitrodibenzofurans (NDBF; yellow), each with very different properties, as summarized in Table 1 ▸ (Corrie *et al.*, 2005 ▸; Mayer & Heckel, 2006 ▸; Hagen, Dekowski *et al.*, 2005 ▸; Klán *et al.*, 2013 ▸; Kaplan *et al.*, 1978 ▸; Cepus *et al.*, 1998 ▸; Barth *et al.*, 1997 ▸; Wieboldt *et al.*, 1994 ▸; Kaplan & Ellis-Davies, 1988 ▸; Ellis-Davies *et al.*, 1996 ▸; Zaitsev-Doyle *et al.*, 2019 ▸; Momotake *et al.*, 2006 ▸; Breitinger *et al.*, 2000 ▸; Monroe *et al.*, 1999 ▸; Bernardinelli *et al.*, 2005 ▸). Full structures of the compounds are shown in Fig. 3 ▸, highlighting the points of photocage attachment/photocleavage.

## 4. The aspects governing the design of a photocaged system   

For every new fast, time-resolved, single-turnover experiment, the characteristics of the photocage to be used have to be tailored. All of these characteristics are intrinsically correlated, and always depend on the photocaging moiety and the substrate being released during cleavage. In general, tailoring one of these aspects causes concurrent changes in all of the others. This correlation is highlighted in Fig. 2 ▸, where the properties of well characterized photocaged compounds can be directly compared. The final photocleavage rates and yield are further modulated by the sample conditions, including the buffer pH and composition.

### 4.1. Photo-decaging rate (*k*)   

**Figure 4:**
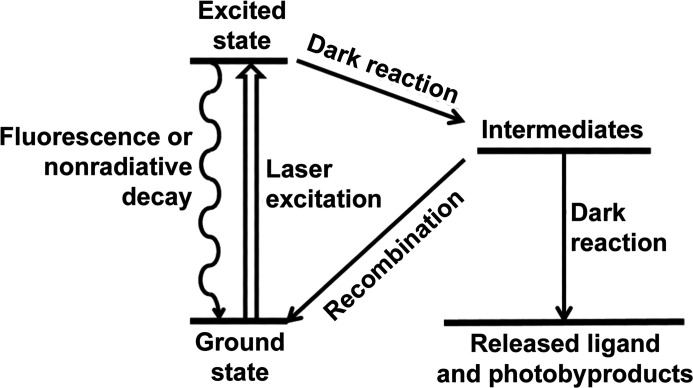
The chemistry of photocleavage. Upon illumination, the photocages undergo a ‘light transition’ into an excited state. Nonproductive events, such as fluorescence or nonradiative decay, can bring the molecule back to the ground state, greatly decreasing the quantum yield of cleavage. The dark reaction proceeds from the excited state through one or more intermediates. The dark reaction determines the rate of compound release and therefore the achievable time resolution.

The photo-decaging rate determines the speed of release of the active species from the photocaging group. It is dependent on the rate of the chemical reaction (bond cleavage and dissociation) that proceeds after the absorption of a photon. As shown in Fig. 4 ▸, these steps are called the ‘dark reaction’, as they are light-independent, and differ considerably between different photocaging groups. *o*NB groups undergo a multi-step dark reaction, generating several metastable distinct intermediates and rearrangements, which are responsible for their relatively slow cleavage rates. In contrast, coumarins undergo a single heterolytic bond scission (fast, hundreds of picoseconds) followed by dissociation of the resulting ion pair and quenching (rate-limiting step, tens of nanoseconds). *p*HP groups undergo a Favorskii rearrangement through a cyclopropanone intermediate, which concurrently releases the ligand in nanoseconds. This property is therefore one of the main determinants of the achievable time resolution of the experiment. It is important to note that other experimental factors, independent of the photocaging group, can ultimately determine the achievable time resolution of an experiment. For example, in a typical monochromatic synchrotron serial crystallography experiments, microcrystals often require several milliseconds of X-ray exposure before a suitable diffraction pattern with sufficient resolution is collected.

### 4.2. The absorption spectrum (λ) and extinction coefficient (ɛ)   

**Figure 5:**
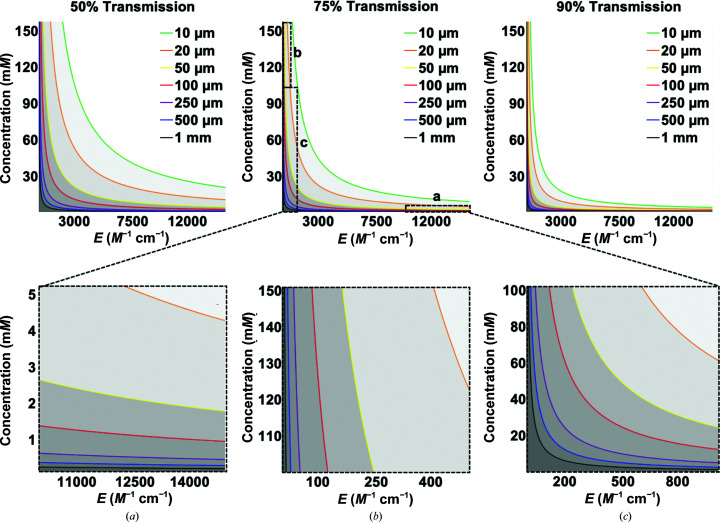
The correlation between sample thickness, concentration and extinction coefficient and their effect on light transmission. The figure shows the 50%, 75% and 90% transmission thresholds of light through samples of varying thickness (10 µm to 1 mm, color-coded). These transmission thresholds correspond to 50%, 25% and 10% attenuation, respectively. The extinction coefficient (*x* axis) and sample concentration (*y* axis) are varied. Insets (*a*)–(*c*) show different areas in more detail for the 75% transmission plot, where (*a*) is high ɛ and low concentration, (*b*) is low ɛ and high concentration and (*c*) is low ɛ and low concentration.

These two quantities are intrinsically correlated and determine the appropriate wavelength of cleavage and the laser power needed to deliver enough photons while keeping a uniform activation throughout the sample thickness. The four photocaging groups discussed here have significantly different absorption profiles. Within the classes, changes to the aromatic moieties further modulate these properties (while also affecting the quantum yield). In general, photoactivation should be carried out at wavelengths of >300 nm to avoid absorption by the protein. Higher extinction coefficients at a given wavelength yield a stronger interaction with light and thus lower laser power is needed for sufficient photon absorption. On the other hand, with concentrated or thick samples the laser light will be highly filtered, with photons being absorbed mostly at the surface of the sample facing the laser, which will lead to non-uniform activation due to attenuation. Fig. 5 ▸ demonstrates the correlation between the extinction coefficient, sample concentration and transmission through different sample thicknesses. The graph shows the maximum concentration of a species with a given extinction coefficient at which the input light is 50%, 25% or 10% attenuated when penetrating samples of different thicknesses (colored contours). As shown, thin samples allow a wide range of concentrations to be used over very different extinction coefficients. In contrast, the useable ranges are very limited for thicker or highly concentrated samples, as light is highly filtered while traveling through the sample. It is worth noting that the typical concentration of proteins in crystals ranges between 5 and 50 m*M*, and depending on the wavelength chosen for activation, thin/microcrystals may have to be used for efficient and uniform illumination. Small crystals have lower diffraction power and may require ultrabright X-ray sources (such as XFELs) for data collection. For diluted solution-phase samples, the experimental design can be more easily tuned, although in many cases sample thicknesses of up to 1 mm are routinely used to obtain sufficient signal-to-noise ratios at synchrotrons. Caged compounds exhibiting low extinction coefficients can be very useful, as described in Section 7, but higher laser powers are required to provide sufficient absorbed photons. In summary, a compromise between extinction coefficient, sample concentration, sample depth and photons deposited has to be achieved to obtain a homogeneous activation through the sample.

### 4.3. Quantum yield (φ)   

Quantum yield is the fraction of molecules that cleave upon absorption of a photon. In most cases, as shown in Fig. 2 ▸, this value is well below 1, typically ranging between 0.1 and 0.4. With a highly *soluble* caged ligand (solubility being another parameter to be considered during photocage design), a high concentration of protected ligand can be introduced either by soaking or during crystallization. The higher concentration can compensate for lower cleavage yields (while accounting for absorption). Depending on the photocaging approach, the ligand may or may not be bound to the protein and so may be ordered or disordered within the crystal lattice. When disordered, the ligand will sit within the bulk solvent in solvent channels, where the diffusion to the active site is of the order of nanometres and therefore occurs on a nanosecond timescale. Disordered photocaged ligands are the most common situation, as photocaging will in many cases target a functionally relevant part of the ligand which is also required for ligand binding. This is not an absolute case, and some systems may allow the introduction of the photocage at a position that arrests activity while not abolishing binding completely. However, very importantly, if the photocaged ligand binds selectively or if the photocage is introduced as a protecting group on the protein itself (through the incorporation of an unnatural amino acid or as a modification post-purification), only a fraction of the sample will be activated upon illumination. This fraction is solely determined by the quantum yield of cleavage. Knowing the quantum yield in advance, the data interpretation can take into account the unactivated fraction, but care has to be taken to obtain sufficient signal-to-noise to accurately distinguish and model the populations.

The question of how much data is needed per time point is not simply answered. It depends on, among other things, the type of structural changes that the experiment is aiming to resolve, the degree of reaction initiation and the quality of the crystals. In practice, data sets of 5000 to 10 000 lattices where the data are of high quality, and the degree of reaction initiation is moderate, are usually sufficient to allow both stable data processing and the observation of structural changes in the resulting electron-density maps. However, we would strongly encourage the collection of as much data as possible for each time point. Bitter experience has shown that it is better to collect fewer time points but ultimately have high-quality electron-density maps than to collect many time points where the data quality is too poor to reveal structural changes. Gorel and coworkers have addressed best practices and provided guidelines for data collection and analysis in XFEL experiments, including time-resolved experiments (Gorel *et al.*, 2021 ▸).

### 4.4. Stability of the final compound and synthetic amenability   

Two further characteristics also have to be examined: synthetic amenability and the stability of the final photocaged compound (*i.e.* the propensity for hydrolysis in the dark). Typically, efficient synthetic routes can be designed by coupling the syntheses of the ligand and the protecting group. In many cases, several synthetic steps are necessary as suitable intermediates are not commercially available. Once synthesized, the stability of the compounds in aqueous solution, and specifically in the buffers and crystallization conditions employed for the final experiment, should be tested.

It is especially important to note the role of buffers. Dark hydrolysis side reactions are generally highly dependent on the pH, as are the cleavage rates of *o*NB groups. The dissociation of ion pairs, such as those in coumarin cleavage, are highly dependent on viscosity. Therefore, for each new compound synthesized, the quantum yield, rate of hydrolysis and extinction coefficient should ideally be determined in the same buffer as will be used for the final time-resolved experiment. Absorption spectra (and therefore extinction coefficients) can be easily obtained using simple laboratory spectrophoto­meters. For quantum yields, typically long-term, low-power illumination is used, and the percentage cleavage is compared with that of a previously characterized compound (using liquid chromatography, for example). To obtain accurate rates of reaction, time-resolved pump–probe spectroscopy using specialist equipment is required.^2^

Nevertheless, as an initial guideline for the design of compounds, the behavior of the families of photocages currently used in biological applications can be generalized. Table 1 ▸ gives a general overview of the behavior of four of the main photocage scaffolds used in fast time-resolved biological studies.

## 5. Photocaging groups and properties   

### 5.1. *Ortho*-nitrobenzyl cages   

Table 2 ▸ shows a summary of *o*NB photocage properties. *o*NB groups are some of the slowest photocleaving moieties, releasing molecules on the greater than tens of milliseconds timescale, but, as shown, changes to the ring substituents as well as at the α-carbon position can change the cage properties quite dramatically. The simplest members of this family, *o*NB and nitrophenyl ethyl (NPE) cages (Figs. 2 ▸ *a* and 2 ▸ *b*, respectively), have short absorption wavelengths (260 nm) and high extinction coefficients at this wavelength. Nevertheless, the absorption spectrum extends well into the 360 nm range, so decaging can be performed at longer wavelengths at the cost of much lower absorption cross sections. The main difference between these two groups is the quantum yield, which is greatly increased from 0.19 to 0.63 between *o*NB and NPE. This increase in quantum yield is caused by the generation of a stable ketone byproduct in NPE cleavage compared with an aldehyde from *o*NB. The absorption profile together with the extinction coefficient can be modulated by the addition of electron-donating groups to the aromatic ring (Fig. 2 ▸ *c*; Corrie *et al.*, 2005 ▸; Klán *et al.*, 2013 ▸). The addition of OMe groups increases the λ_max_ from 280 to 355 nm, with a severe decrease in both ɛ (from 27 000 to 5000 *M* ^−1^ cm^−1^) and φ (from 0.63 to 0.07; Figs. 2 ▸ *b* and 2 ▸ *c*). One subset of these cages, carboxy­nitrobenzyls (CNBs), can reach faster cleavage rates, in the hundreds of microseconds, by the addition of a carboxylate at the α position (Fig. 2 ▸ *f* versus Fig. 2 ▸ *j*). The carboxylate also provides a better solubility profile to the photocage, but presents some synthetic challenges, especially if a single diastereoisomer is required. The combination of the two factors (electron-donating groups at the ring and introduction of the α-carboxylate) yields a cage with mixed properties: a longer λ_max_ and a higher solubility, but a slightly slowed cleavage rate and extinction coefficient (Fig. 2 ▸ *k*). Cleavage rates are also highly dependent on the nature of the leaving group (Fig. 2 ▸ *c* versus Fig. 2 ▸ *g* and Fig. 2 ▸ *j* versus Fig. 2 ▸ *l*).

### 5.2. *Para*-hydroxyphenyl cages   

*p*HPs have fast cleavage rates (nanosecond timescales), but they also present a low λ_max_ (∼280 nm), which can be difficult to modulate without altering their other properties. A simple comparison between *p*HP-ATP and NPE-ATP (Figs. 2 ▸ *e* and 2 ▸ *b*) shows the clear difference in solubility and cleavage rate between the two compounds, with similar properties otherwise. Comparing *p*HP-ATP and *p*HP-Glu (Figs. 2 ▸ *e* and 2 ▸ *i*) shows little difference in properties associated with a change in leaving group, in contrast to the large differences observed for the same compounds protected by NPE groups (Figs. 2 ▸ *b* and 2 ▸ *f*).

The addition of electron-donating groups (for example OMe groups; Table 3 ▸) increases the absorption wavelength at a severe cost to quantum yield. Nevertheless, these cages are small and easy to synthesize and confer good solubility compared with other photocaging scaffolds. Interestingly, a recently reported time-resolved diffraction experiment driven by *p*HP-fluoroacetate decaging reported cleavage of the cage at >300 nm (see Section 7; Mehrabi *et al.*, 2019 ▸). In this case, the absorption band of the photocage was red-shifted when soaked into the protein crystals. This phenomenon highlights the importance of testing the spectroscopic and decaging properties of compounds in the same environment as used for the final time-resolved experiment. Furthermore, the ionizable character of the phenol group makes the photocage highly dependent on pH, which is accompanied by considerable changes to its absorption spectrum depending on the protonation state (Klán *et al.*, 2013 ▸).

### 5.3. Coumarinyl cages   

Coumarinyl photolysis half-lives are comparable to those of *p*HP groups (∼10 ns) and are therefore faster than nitrodibenzofurans (NDBF) and much faster than *o*NB groups. They have long-wavelength absorption maxima (λ_max_ > 300 nm) and large extinction coefficients (see Table 4 ▸ for general properties). The increased absorption wavelength means that coumarins are well suited as photocages for proteins and protein substrates as well as for nucleic acids. Table 5 ▸ shows a collection of examples of biologically relevant coumarinyl-protected ligands. Even though coumarins have been well characterized spectroscopically, little information has been reported for their photolysis kinetics. From the mechanism of cleavage (homolytic scission of the α-carbon–leaving group bond; Klán *et al.*, 2013 ▸) the typical cleavage rates are ∼1 × 10^8^ s^−1^, easily allowing the study of enzymatic reactions. However, their synthesis can be challenging when compared with *o*NBs and *p*HPs, and they can have poor solubility due to the conjugated aromatic ring system. Hydrophilic and ionizable groups, such as hydroxyls and carboxylates, can be added to increase solubility (see Table 4 ▸).

The λ_max_ is mostly controlled by the substituents at the 6- and 7-positions of the ring. The introduction of electron-withdrawing groups, such as bromine, red-shifts the absorption maximum by ∼50 nm. A similar effect can be observed with the introduction of 7-dialkylamines. In both cases, an increase in the absorption cross section is also reported. The quantum yields for decaging are relatively low, remaining below 0.3, and are highly dependent on the leaving group. The introduction of small concentrations of organic solvents into the mixtures, used to help solubilize the photocaged compounds, can also have an effect on the final quantum yields.

### 5.4. Nitrodibenzofuranyl cages   

NDBFs, as described by Momotake and coworkers, present very desirable properties for the decaging of biologically relevant small molecules (Momotake *et al.*, 2006 ▸). They have been described for the release of ATP and EGTA (Ca^2+^ release). Although NDBFs are not the fastest cages (∼50 µs), they have desirable absorption maxima (330–380 nm). The reported quantum yields vary between 0.2 and 0.7, respectively, for NDBF-cysteine (in a peptide; Mahmoodi *et al.*, 2016 ▸). With large absorption cross sections and faster cleavage rates than *o*NB groups, these cages cover an interesting photochemical space complementary to the other, more commonly used chemical groups.

## 6. Specialty photocages   

### 6.1. Photoacids   

Photoacids are molecules that are converted into strong acids upon irradiation with light (Liao, 2017 ▸). They can be described as irreversible (PAGs), reversible (PAHs) or metastable (mPAHs), and structures of representative photoacids and their properties are shown in Table 6 ▸. Irreversible and reversible photoacids were the focus of early research due to their rapid conversion to the acidic state upon irradiation, giving temporal control over pH-initiated processes on the order of nanoseconds. The PAG 2-nitrobenzaldehyde has been used to probe the pH dependence of acid phosphatase activity in solution with a time resolution of 250 ms, using a 388 nm pulsed laser (Kohse *et al.*, 2013 ▸). In turn, pyranine, a PAH, was employed in a time-resolved fluorescence study to determine the optimal drug–gel match in a drug-delivery hydrogel (Nandi *et al.*, 2020 ▸). Although they remain useful in pH-jump experiments, PAGs lack the ability to return to the less acidic excited state, and early PAHs lack the ability to create pH jumps of larger than 1–2 units due to rapid conversion back to the non-acidic state (Berton *et al.*, 2020 ▸). Liao and coworkers described the first metastable photoacids (mPAHs), allowing more pronounced, reversible pH jumps (up to six units) with irradiation at wavelengths longer than 400 nm in water (Liao, 2017 ▸). Merocyanine (and its derivatives) are the most widely studied mPAHs and have high quantum yields (φ = 0.7 for merocyanine itself; Berton *et al.*, 2020 ▸).

### 6.2. Molecular photoswitches   

Photoswitches differ from photocages in that they change conformation (usually through either *cis*–*trans* isomerization or by transitioning from closed to open forms) rather than undergoing covalent bond cleavage with release of a product upon light activation (Szymański *et al.*, 2013 ▸). Such switches can be found in a number of naturally photoactivatable proteins, such as PYP (Pande *et al.*, 2016 ▸), rhodopsin (Asido *et al.*, 2019 ▸) and fluorescent proteins (Woodhouse *et al.*, 2020 ▸). Azobenzene is by far the best studied and most commonly used non-natural photoswitch. *Trans*–*cis* isomerization is triggered by the absorption of light at 320 nm and *cis*–*trans* isomerization happens either thermally or from absorption of visible light (>460 nm). Typical chromophore isomerization rates are subpicosecond and thermal relaxation half-lives in the multiple seconds to hours regime, depending on the ring substituents and the solvent (Renner & Moroder, 2006 ▸; Zhu & Zhou, 2018 ▸). Quantum yields are very high, as there are virtually no competing photochemical reactions to the isomerization (Kumar & Neckers, 1989 ▸). Stilbenes are similar to azobenzenes. They undergo a *trans*–*cis* isomerization promoted by UV irradiation (>313 nm, depending on the substituents; Szymański *et al.*, 2013 ▸) and have high quantum yields (φ = 0.5; Waldeck, 1991 ▸). However, stilbenes can undergo oxidation/cyclization in the *cis*-conformation, causing reversibility issues. Spiropyrans and diarylethenes are examples of photoswitches that undergo ring opening/closing through bond scission rather than isomerization. For spiro­pyrans, bond scission is triggered by UV radiation (365 nm) and re-cyclization by visible irradiation (>460 nm). The opposite is observed with diarylethenes, which cyclize upon UV radiation and ring-open with visible light.

Molecular photoswitches have been introduced into a diverse set of biomolecules to drive conformational changes and observe the induced dynamics. Studies have included the photoswitching of nucleic acids, for example by stapling ssDNA through backbone cross-linking and controlling hairpin stability with light (Lewis *et al.*, 2002 ▸). Ultrafast spectroscopy of small peptide folding has revealed insights into protein-folding mechanisms on ultrafast timescales (Renner & Moroder, 2006 ▸). One application of specific interest for time-resolved structural biology is the covalent linkage of ligands to proteins through a photoswitchable linker. Light-induced isomerization of the ligand promotes binding or release of the ligand and downstream activation of the protein. Trauner and coworkers demonstrated this approach using two transmembrane proteins: a glutamate receptor and a voltage-gated potassium ion channel (Volgraf *et al.*, 2006 ▸; Kramer *et al.*, 2005 ▸). There are many other examples of such studies, which have been comprehensively reviewed elsewhere (Szymański *et al.*, 2013 ▸; Zhu & Zhou, 2018 ▸).

### 6.3. Metal-containing photocages   

Metal-containing photolabile protecting groups expand the scope of molecules that can be released with light beyond the typical good leaving groups presented in the earlier sections of this review. Furthermore, many absorb strongly at longer wavelengths, allowing cleavage with visible and near-infrared light.

**Figure 6:**
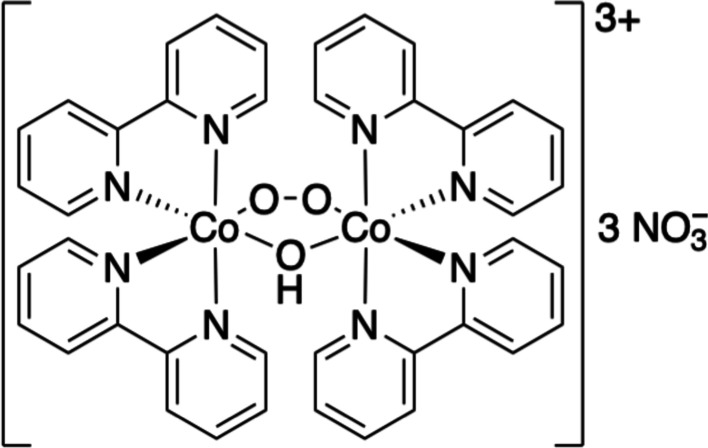
Cobalt-based O_2_ photocage.

Dioxygen is possibly one of the most important molecules that can be photocaged using metal-containing photocages. Cobalt-containing peroxo photocages are one such example (Ludovici *et al.*, 2002 ▸). These compounds (Fig. 6 ▸) are soluble in aqueous buffers and have been shown to release O_2_ by illumination at wavelengths ranging between 290 and 390 nm, yielding quantum yields of 0.1–0.5, with a maximum quantum yield achieved at 315 nm. The cleavage rates are also fast (<1 µs) and have been used to study oxygen binding to cytochrome *bo*_3_ using spectroscopy (Ludovici *et al.*, 2002 ▸). The photocage can also be cleaved at cryogenic temperatures, trapping dioxygen, which can be used to trigger subsequent reactions by increasing the temperature (Howard-Jones *et al.*, 2009 ▸). Although not a time-resolved experiment in the typical sense of the technique, this approach allows intermediates to be generated and captured by cryo-quenching. Of course, the use of such photocages requires sample preparation under strict anaerobic conditions.

Metal-containing photocages can also release both the metal center and the ligands coordinated to it, allowing complementary effects to be studied and action as so-called dual-action agents (Havrylyuk *et al.*, 2020 ▸). Such photocages are useful for the study of metals, as well as nitriles, thioethers and aromatic heterocycles that can be typically found as part of protein mechanisms but which cannot be caged using the more traditional photocages.

Ruthenium(II) polypyridyl complexes are the most commonly used molecules in metal-based biorelevant photocages (Li *et al.*, 2018 ▸). Different ligands offer different photophysical properties, reduced cytotoxicity and increased stability. Ruthenium(II) complexes bearing bidentate ligands (such as compound **1** in Table 7 ▸) have been shown to have modest stability (∼40% hydrolysis in water at 37°C in 24 h) and quantum yields of 0.14 at 510 nm irradiation. Other similar ruthenium(II) compounds (Table 7 ▸, compounds **2** and **3**) release nitrile groups upon irradiation at 350–400 nm with quantum yields of 0.01–0.03, and variations of such compounds bearing nitriles or nicotines can be cleaved with higher quantum yields (φ = 0.2) when irradiated with 400–470 nm light (Li *et al.*, 2018 ▸; Filevich *et al.*, 2010 ▸).

Other metal complexes can also be used for photocaging. For example, zinc complexes (Table 7 ▸, compound **4**) can be cleaved using 365 nm illumination with quantum yields of 0.27, releasing Zn^2+^ and CO_2_ (Basa *et al.*, 2015 ▸, 2019 ▸). Rhodium(III) and copper(II) complexes have been shown to bind DNA and release it with light of 455–610 nm wavelength for rhodium(III) and 312–694 nm wavelength for copper(II) (Schatzschneider, 2010 ▸). Other DNA photocages include copper(II), nickel(II) and zinc(II) complexes bearing dpq ligands (Table 7 ▸, compound **5**). These bind DNA at the minor groove and release it upon illumination at 340–345 nm (2100–3820 *M* ^−1^ cm^−1^). Their absorption cross section tails well into the 500–670 nm range, depending on the metal center (15–105 *M* ^−1^ cm^−1^; Roy *et al.*, 2008 ▸).

## 7. Three examples of the use of photo-decaging for time-resolved structural biology with X-rays   

There have been some recent publications demonstrating the use of photo-decaging strategies to trigger protein function in time-resolved structural biology experiments with X-ray radiation. Here, we highlight three of these studies.

The first study looked at the dimerization of soluble nucleotide-binding domains (NBDs) of the bacterial lipid flippase MsbA (Josts *et al.*, 2018 ▸). In these experiments, NPE-ATP (Fig. 2 ▸ *b*) was used as a light-deprotectable ATP analog. Upon irradiation, the NDBs bind ATP and subsequent dimerization occurs. X-ray solution scattering was used to probe the transition. Although NPE-ATP has a relatively slow release time (∼10 ms), it was sufficiently fast to capture the dimerization step, which occurred with a rate of 6200 *M* ^−1^ s^−1^ in combination with a high quantum yield of 0.6, which allowed a rapid increase of ATP concentration *in situ*. To obtain good scattering profiles, a 1 mm sample capillary was used, and so the low ɛ of NPE at the chosen laser wavelength (355 nm) allowed full capillary penetration and uniform decaging across the sample. This is an example where a lower absorption cross section was vital to yield a uniform activation.

A second example was described by Mehrabi and coworkers, in which time-resolved diffraction was used to follow the catalytic turnover in fluoroacetate dehalogenase (FAcD) from *Rhodopseudomonas palustris* (Mehrabi *et al.*, 2019 ▸). The study revealed the mechanism of action of the enzyme by crystallography as well as correlated inter-subunit breathing motions that were not previously observed. In this experiment, FAcD microcrystals were soaked with *p*HP-caged fluoroacetate. Normally, *p*HP cages have narrow absorption profiles, peaking at 280 nm in solution. Surprisingly, however, when soaked into the protein crystals, the absorption band was red-shifted, allowing decaging with a 344 nm laser, well away from the protein absorption bands. In this experiment, the caged ligand was not bound in the enzyme active site but was present in the solvent channels in the crystal. This allowed the release of excess substrate (4–14 times the protein concentration in the crystal, estimated from the initial ligand concentration and the expected quantum yield) which could then diffuse rapidly to the active sites, promoting complete activation of the protein molecules. If an active-site residue had been photocaged instead, the final fraction of active molecules contributing to the signal would have been dependent only on the quantum yield of decaging (10–40%). This low percentage of activation would likely have required the acquisition of considerably more highly redundant data to reliably visualize the structural changes associated with catalysis.

The third example we highlight makes use of nitric oxide (NO) photocaging (Tosha *et al.*, 2017 ▸; Nomura *et al.*, 2021 ▸). Nomura and coworkers and Tosha and coworkers employed a water-soluble NO photocage that demonstrates fast cleavage rates (tens of microseconds) and high quantum yields (1.98, as one molecule can release two NO molecules) at 308 nm illumination (Namiki *et al.*, 1997 ▸). P450nor crystals soaked with photocaged NO and illuminated at 308 nm allowed transient structures of the NO-bound intermediate to be obtained after a 20 ms time delay using serial femtosecond crystallography. The illumination parameters had to be tuned to avoid protein damage while still allowing sufficient decaging. Due to this necessity, only 50% of the sites were occupied with NO. Nevertheless, the structure is clear, especially due to the collection of concurrent dark frames that afforded a very well defined ground-state structure for modeling. The modeled species (ferric NO) was corroborated by UV–Vis and IR spectroscopy, and low-dose cryocrystallography, all performed with microcrystals. One interesting aspect of this study is a comparison of spectroscopic data from protein in solution and in microcrystals, which revealed a 20-fold decrease in reaction rate, possibly due to restrained movement in the lattice. This prior knowledge provided vital insight for the correct design of the diffraction experiments as well as validation for the final proposed protein model.

## 8. Conclusion   

Although photocaging chemistry has been explored for several decades, its use in fast, single-turnover biophysical experiments is still severely underdeveloped. The main reason is that the time-resolved spectroscopic experiments needed to characterize the decaging rate of such compounds are complex to perform. Nevertheless, some general trends in the behavior of common photocaging groups can be established and their properties extrapolated. The guidelines presented here are to be used as an initial approach for the choice of the correct protecting group for a given time-resolved experiment which satisfies the different requirements: extinction coefficient, absorption maximum, quantum yield and cleavage rate. The chosen cage should, of course, always be characterized with ultrafast spectroscopy before the final diffraction/scattering experiment. It is important to note that most cages are designed to release good leaving groups (for example thiols, alcohols and carboxylates) and that the timescales and quantum yields tend to decrease considerably when other functional groups are caged. Although still in its infancy, the use of photocaging for time-resolved X-ray studies is opening the way to the study of more complex biological targets, while also complementing sample-delivery methods such as viscous jets and fixed targets which are less amenable to rapid mixing. One further application of photocaging for structural biology relies on the rapid cryocooling of samples. In this case, the sample is photoactivated and quickly cryo-quenched to deliver a vitrified arrested state at a specific time delay from photocleavage. Such approaches are expanding the use of photocages to time-resolved cryo-EM by using a light-coupled cryo-plunger (Yoder *et al.*, 2020 ▸).

## Figures and Tables

**Table 1:** General properties of the four photocaging scaffolds discussed in this review R stands for different chemical groups that can be added to the overall scaffold. LG stands for leaving group: the molecular fragment released after photoexcitation (Goeldner & Givens, 2006 ▸; Klán *et al.*, 2013 ▸; Mahmoodi *et al.*, 2016 ▸; Momotake *et al.*, 2006 ▸; Becker *et al.*, 2018 ▸).

				
	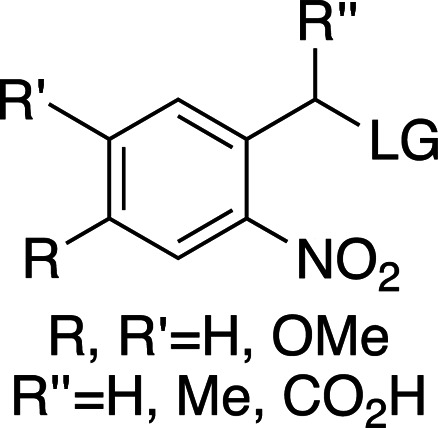	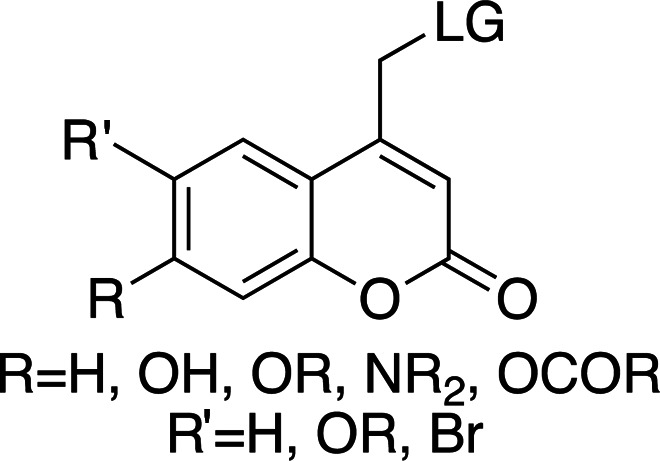	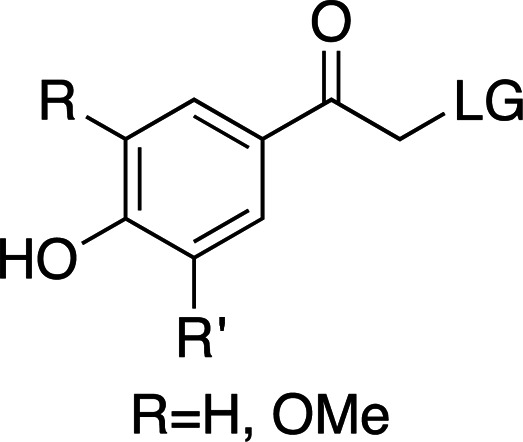	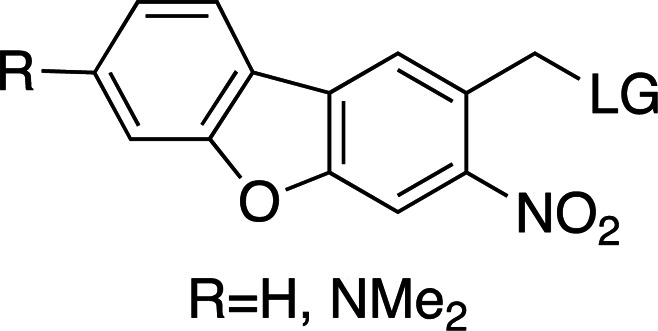
Group	*ortho*-Nitrobenzyl	Coumarinyl	*para*-Hydroxyphenyl	Nitrodibenzofuranyl
λ_max_ (nm)	254–345	320–390	280–304	310–420
ɛ_max_ (*M* ^−1^ cm^−1^)	600–27000	6000–20000	9000–15000	10000–19000
Φ†	0.01–0.64	0.02–0.30	0.03–0.9	0.2–0.7
*k* (s^−1^)	10 to 3 × 10^4^	1 × 10^8^ to 2 × 10^9^	1 × 10^7^ to 2 × 10^9^	2 × 10^4^
Solubility (H_2_O)	Poor–medium	Poor–medium	Good	Poor–medium

† The range of values is quoted as a trend relative to the range of λ_max_. Φ tends to decrease with increasing λ_max_ for *o*NB and *p*-hydroxyphenyl groups and tends to increase with λ_max_ for coumarins.

**Table 2:** General properties of *o*NB photocages with different ring and α-carbon substituents The ligand released by photocleavage is represented by LG.

				
	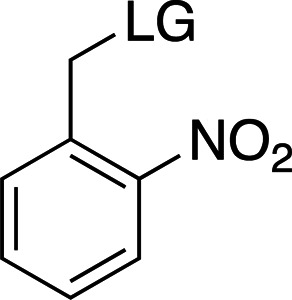	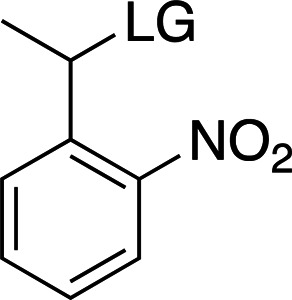	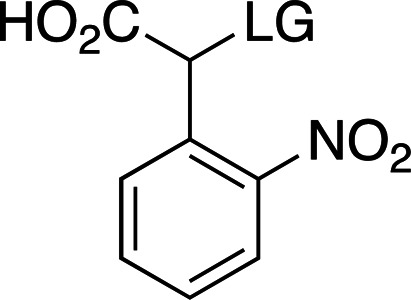	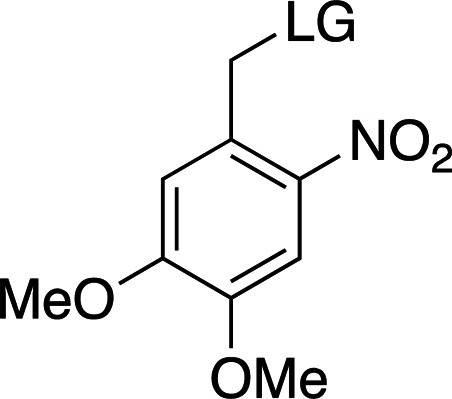
λ_max_ (nm)	254	254	262	345
ɛ_max_ (*M* ^−1^ cm^−1^)	∼27000	∼27000	∼5000	∼6000
φ	0.1–0.2	0.1–0.64	0.04–0.14	0.01
*k* (s^−1^)	10–200	10–1000	9 × 10^3^ to 3 × 10^4^	N/A
Solubility (H_2_O)	Poor	Poor	Good	Poor
Reference	Goeldner & Givens (2006 ▸)	Goeldner & Givens (2006 ▸)	Grewer *et al.* (2000 ▸)	Aujard *et al.* (2006 ▸)

**Table 3:** General properties of *p*HP photocages, highlighting the effect of adding electron-donating groups to the ring (Klán *et al.*, 2013 ▸) The ligand released by photocleavage is represented by LG.

			
	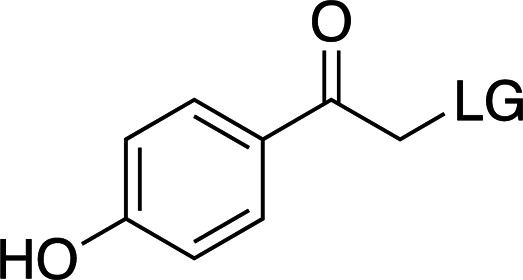	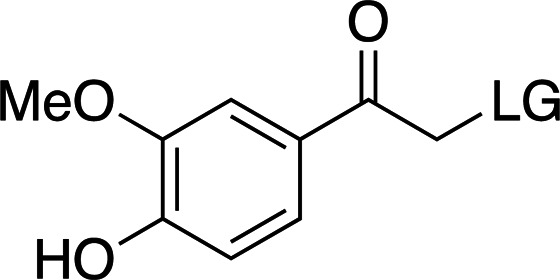	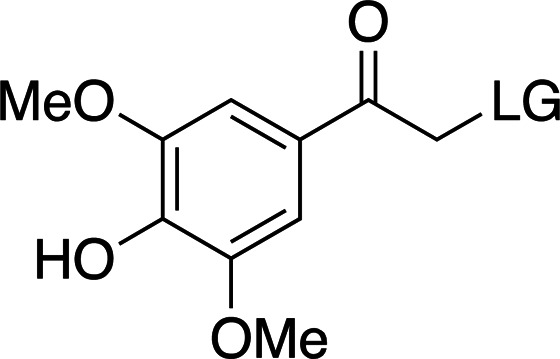
λ_max_ (nm)	285	280	304
ɛ_max_ (*M* ^−1^ cm^−1^)	∼15000	∼9000	∼12000
φ	0.2–0.9	0.03–0.04	0.03
*k* (s^−1^)	1 × 10^7^ to 2 × 10^9^	∼2 × 10^9^	∼2 × 10^7^
Solubility (H_2_O)	Good	Good	Good

**Table 4:** General properties of coumarin-caged compounds, highlighting the effects of ring substituents (Goeldner & Givens, 2006 ▸) For most, no information on cleavage kinetics has been reported. The ligand released by photocleavage is represented by LG.

				
	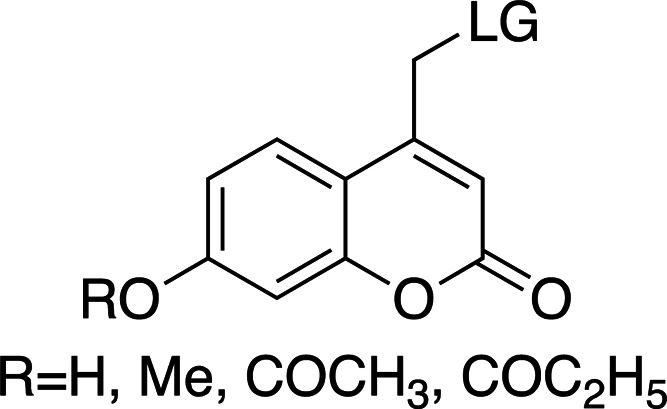	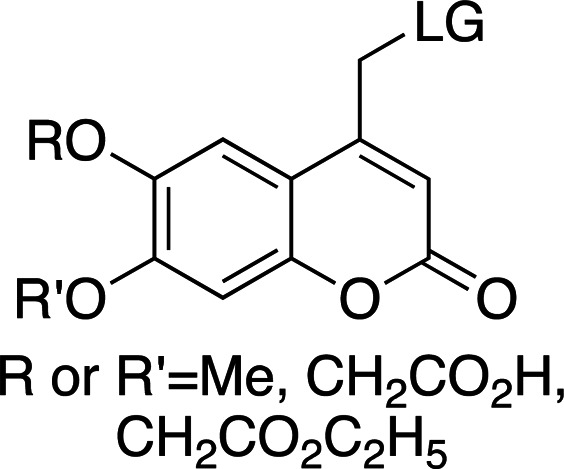	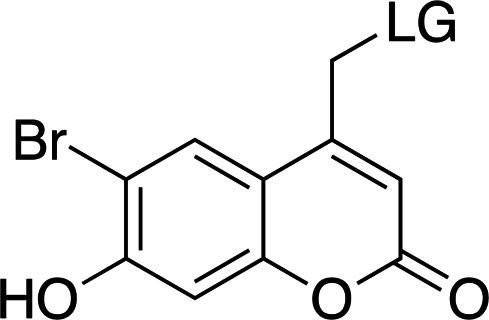	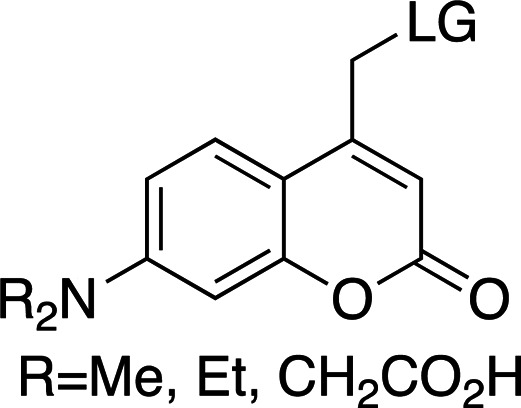
λ_max_ (nm)	320–330	345	370–380	380–390
ɛ_max_ (*M* ^−1^ cm^−1^)	6000–13300	1000–12000	13000–19000	15000–20000
φ	0.02–0.15	0.04–0.1	0.02–0.11	0.09–0.3†
*k* (s^−1^)	1 × 10^8^ to 4 × 10^8^	—	—	1 × 10^9^ to 2 × 10^9^
Solubility (H_2_O)	Low	Low/moderate‡	Low	Low/moderate‡
References	Furuta & Iwamura (1998 ▸), Furuta *et al.* (1999 ▸)	Furuta & Iwamura (1998 ▸), Furuta *et al.* (1999 ▸)	Furuta *et al.* (1999 ▸)	Geissler *et al.* (2003 ▸), Hagen *et al.* (2001 ▸), Hagen, Dekowski *et al.* (2005 ▸)

† Some studies wre in water/solvent mixtures (20% methanol and 5% acetonitrile), where the presence of organic solvents may increase the quantum yield.

‡ Increased solubility with methylcarboxylate (–CH_2_CO_2_H) substituents.

**Table 5:** Overview of typical photoacids and their properties

			
	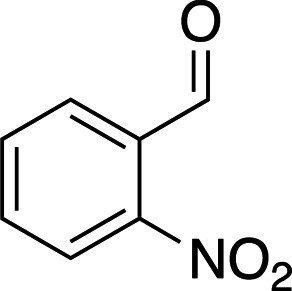	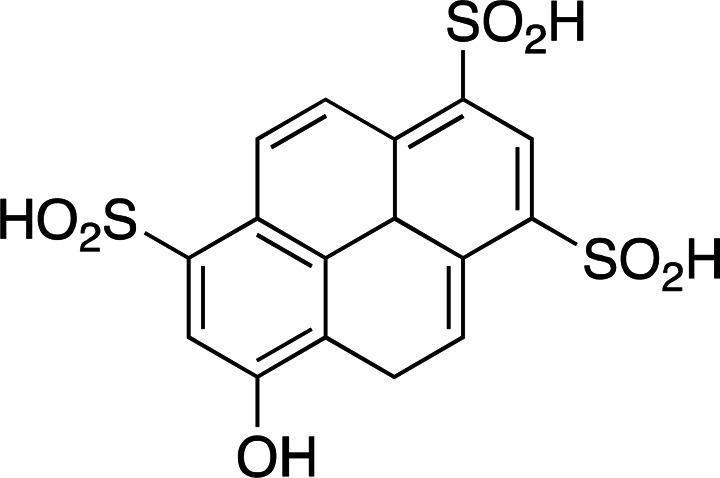	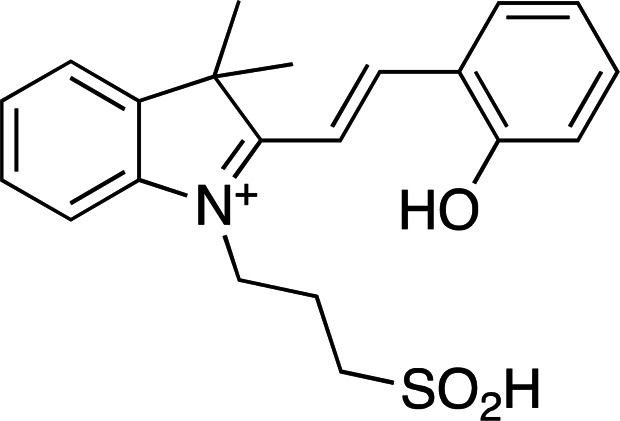
Photoacid name	2-Nitrobenzaldehyde	Pyranine	Merocyanine
Photoacid type	PAG	PAH	mPAH
λ_max_ (nm)	355 (H_2_O)	410 (pH 3), 466 (pH 10)	533 (H_2_O)
ɛ_max_ (*M* ^−1^ cm^−1^)	30000 (H_2_O)	8200 (pH 3), 11500 (pH 10)	47000 (H_2_O)
p*K*_a_	2.9 (H_2_O)	7.35 (buffer)	2.14 (H_2_O)
References	Chaves *et al.* (2016 ▸)	Borba *et al.* (2000 ▸)	Dixit & Mackay (1983 ▸), Liao (2017 ▸)

**Table 6:** Photoswitching scaffolds showing the two light-induced conformations and the typical wavelengths for transition

	Azobenzene	Stilbene	Spiropyran	Diarylethene
State 1	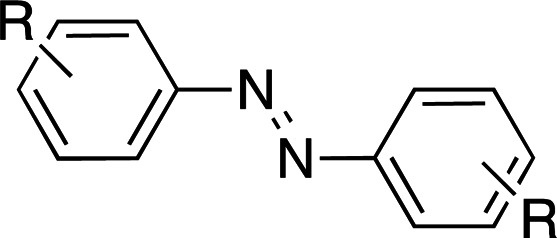	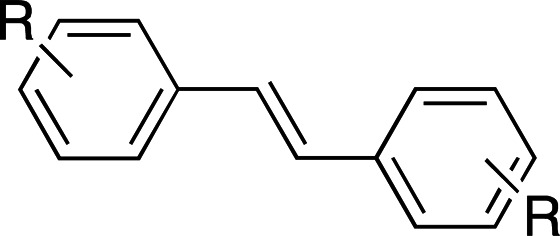	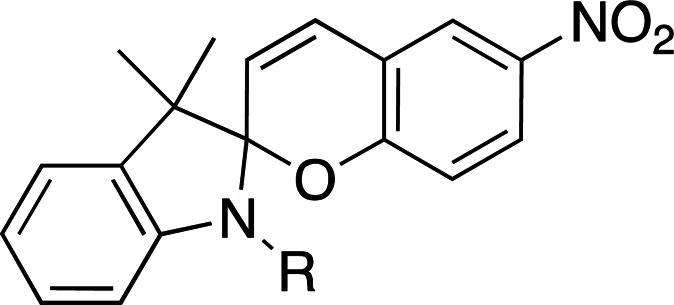	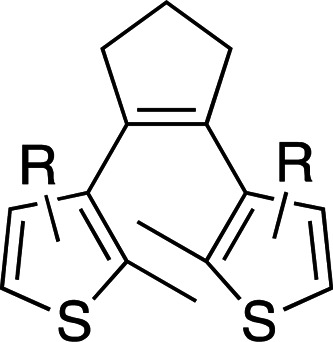
State 2	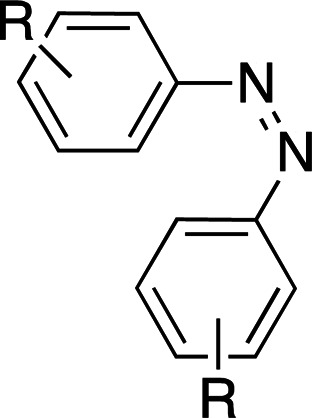	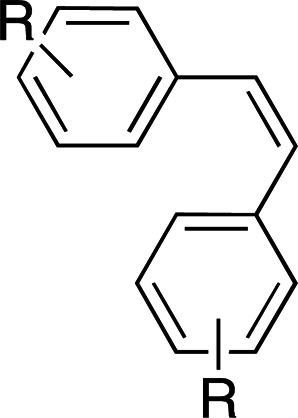	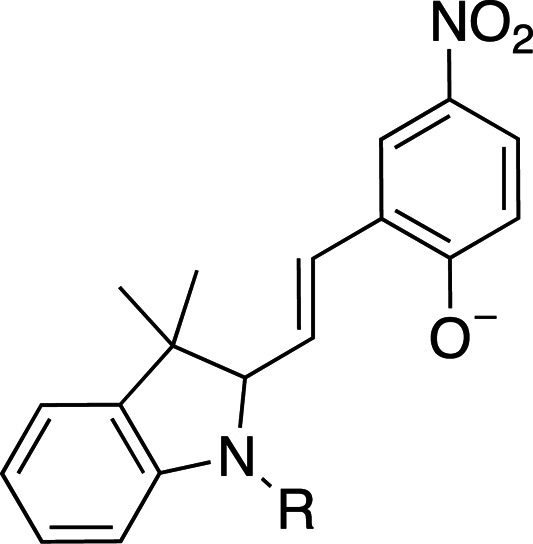	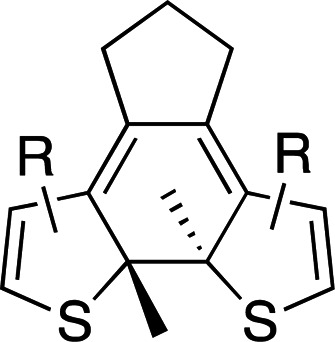
Transitions	320 nm, >460 nm	Both UV (>313 nm, R-dependent)	365 nm, >460 nm	UV, visible (R-dependent)

**Table 7:** Further examples of metal-containing photocages and typical photoactive properties

					
	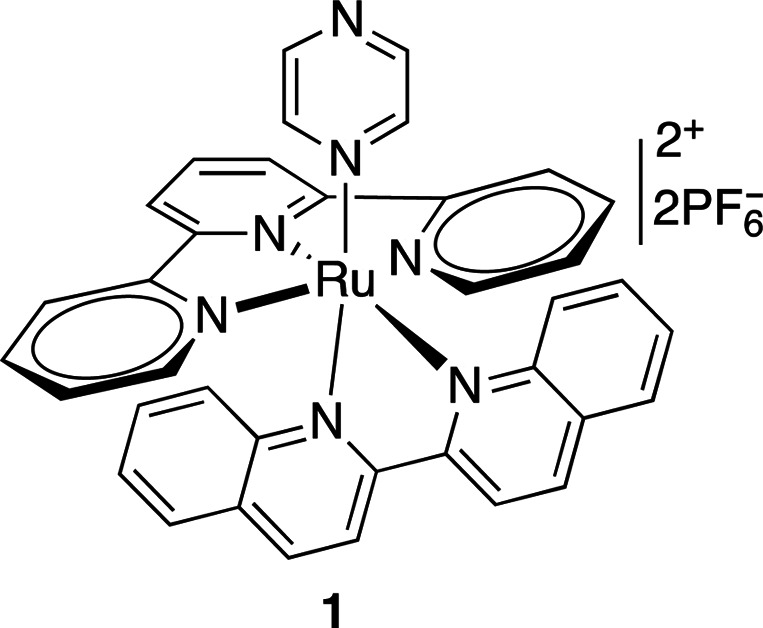	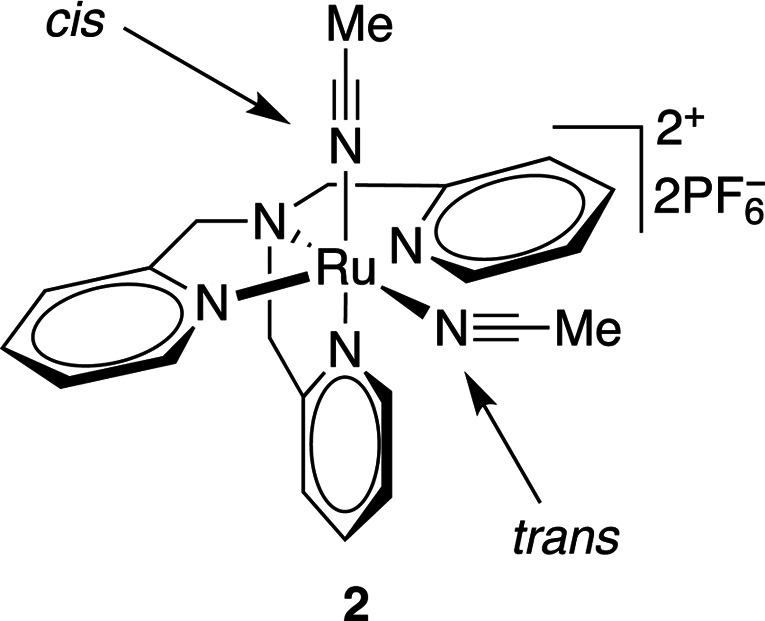	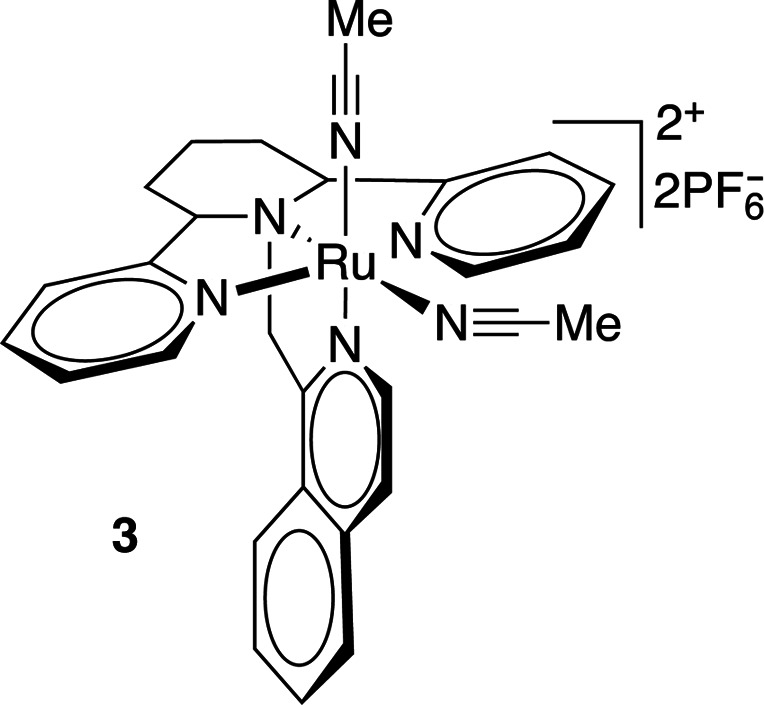	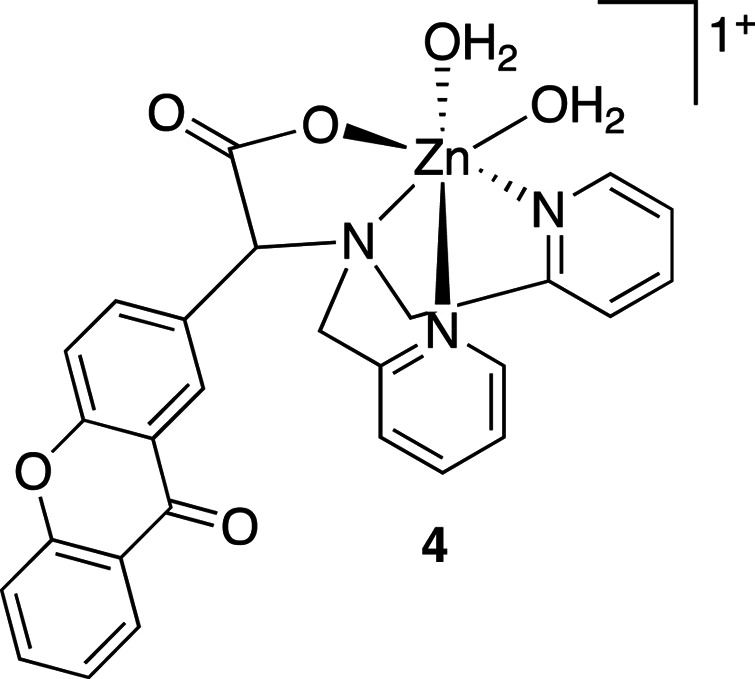	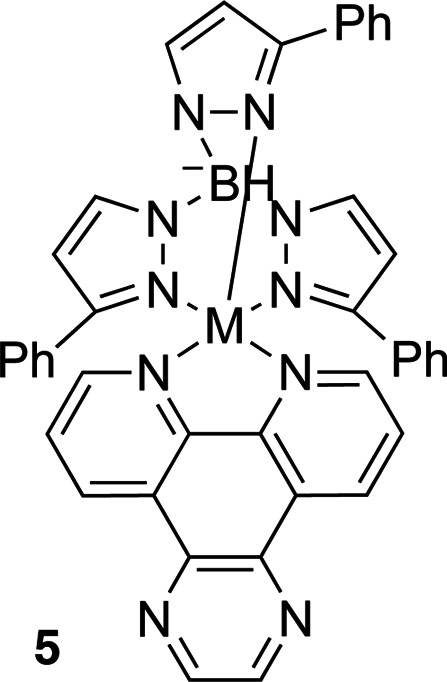
Released ligand	Pyrazine	*cis*-Nitrile	*cis*-Nitrile	CO_2_, Zn^2+^	DNA
λ_max_ (nm)	510	380	390	344	518–643, 340–345
ɛ_max_ (*M* ^−1^ cm^−1^)	9900	11200	11900	5270	15–105, 2100–3820
φ	0.14	0.01	0.03	0.27	—
References	Havrylyuk *et al.* (2020 ▸)	Li *et al.* (2018 ▸)	Li *et al.* (2018 ▸)	Basa *et al.* (2019 ▸)	Roy *et al.* (2008 ▸)

## Footnotes

1 This estimate was first made by M. Thompson (University of California Merced), based on a SWISS–PROT server search for relevant keywords, and was presented at the BioXFEL Annual Meeting in 2017. Repeating this search using the keywords light harvesting, photo, light activated, light sensitive, opsin and chrome yielded 1681 results out of a total of 565 254 entries (on 16 July 2021), equating to 0.3%. We have therefore conservatively estimated <0.5% as a suitable number to account for any relevant entries not identified during this search.

2 There are an increasing number of user facilities offering access to time-resolved spectroscopy. The Laserlab Europe Consortium coordinates access to user facilities across Europe (https://www.laserlab-europe.eu/). In the US there are user facilities at Argonne (https://www.anl.gov/cnm/terahertztoultraviolet-ultrafast-spectroscopy) and Brookhaven (https://www.bnl.gov/cfn/facilities/optics.php) National Laboratories.

## References

[bb1] Aarhus, R., Gee, K. & Lee, H. C. (1995). *J. Biol. Chem.* **270**, 7745–7749. 10.1074/jbc.270.13.7745 7706323

[bb2] Asido, M., Eberhardt, P., Kriebel, C. N., Braun, M., Glaubitz, C. & Wachtveitl, J. (2019). *Phys. Chem. Chem. Phys.* **21**, 4461–4471. 10.1039/c8cp07418f 30734791

[bb151] Aujard, I., Benbrahim, C., Gouget, M., Ruel, O., Baudin, J., Neveu, P. & Jullien, L. (2006). *Chem. Eur. J.* **12**, 6865–6879. 10.1002/chem.200501393 16763952

[bb3] Barth, A., Corrie, J. E. T., Gradwell, M. J., Maeda, Y., Mäntele, W., Meier, T. & Trentham, D. R. (1997). *J. Am. Chem. Soc.* **119**, 4149–4159.

[bb4] Basa, P. N., Antala, S., Dempski, R. E. & Burdette, S. C. (2015). *Angew. Chem. Int. Ed.* **54**, 13027–13031. 10.1002/anie.201505778 PMC4615554 26346802

[bb5] Basa, P. N., Barr, C. A., Oakley, K. M., Liang, X. & Burdette, S. C. (2019). *J. Am. Chem. Soc.* **141**, 12100–12108. 10.1021/jacs.9b05504 31256585

[bb6] Baxter, R. H. G., Ponomarenko, N., Srajer, V., Pahl, R., Moffat, K. & Norris, J. R. (2004). *Proc. Natl Acad. Sci. USA*, **101**, 5982–5987. 10.1073/pnas.0306840101 PMC395909 15073325

[bb7] Becker, Y., Unger, E., Fichte, M. A. H., Gacek, D. A., Dreuw, A., Wachtveitl, J., Walla, P. J. & Heckel, A. (2018). *Chem. Sci.* **9**, 2797–2802. 10.1039/c7sc05182d PMC5914290 29732066

[bb8] Behrens, C., Decker, F.-J., Ding, Y., Dolgashev, V. A., Frisch, J., Huang, Z., Krejcik, P., Loos, H., Lutman, A., Maxwell, T. J., Turner, J., Wang, J., Wang, M.-H., Welch, J. & Wu, J. (2014). *Nat. Commun.* **5**, 3762. 10.1038/ncomms4762 24781868

[bb9] Bernardinelli, Y., Haeberli, C. & Chatton, J.-Y. (2005). *Cell Calcium*, **37**, 565–572. 10.1016/j.ceca.2005.03.001 15862347

[bb10] Berton, C., Busiello, D. M., Zamuner, S., Solari, E., Scopelliti, R., Fadaei-Tirani, F., Severin, K. & Pezzato, C. (2020). *Chem. Sci.* **11**, 8457–8468. 10.1039/d0sc03152f PMC8163397 34123105

[bb11] Beyerlein, K. R., Dierksmeyer, D., Mariani, V., Kuhn, M., Sarrou, I., Ottaviano, A., Awel, S., Knoska, J., Fuglerud, S., Jönsson, O., Stern, S., Wiedorn, M. O., Yefanov, O., Adriano, L., Bean, R., Burkhardt, A., Fischer, P., Heymann, M., Horke, D. A., Jungnickel, K. E. J., Kovaleva, E., Lorbeer, O., Metz, M., Meyer, J., Morgan, A., Pande, K., Panneerselvam, S., Seuring, C., Tolstikova, A., Lieske, J., Aplin, S., Roessle, M., White, T. A., Chapman, H. N., Meents, A. & Oberthuer, D. (2017). *IUCrJ*, **4**, 769–777. 10.1107/S2052252517013124 PMC5668862 29123679

[bb12] Binder, D., Bier, C., Grünberger, A., Drobietz, D., Hage-Hülsmann, J., Wandrey, G., Büchs, J., Kohlheyer, D., Loeschcke, A., Wiechert, W., Jaeger, K., Pietruszka, J. & Drepper, T. (2016). *ChemBioChem*, **17**, 296–299. 10.1002/cbic.201500609 26677142

[bb13] Borba, E. B. de, Amaral, C. L. C., Politi, M. J., Villalobos, R. & Baptista, M. S. (2000). *Langmuir*, **16**, 5900–5907.

[bb14] Bourgeois, D., Vallone, B., Arcovito, A., Sciara, G., Schotte, F., Anfinrud, P. A. & Brunori, M. (2006). *Proc. Natl Acad. Sci. USA*, **103**, 4924–4929. 10.1073/pnas.0508880103 PMC1458771 16547137

[bb15] Breitinger, H. A., Wieboldt, R., Ramesh, D., Carpenter, B. K. & Hess, G. P. (2000). *Biochemistry*, **39**, 5500–5508. 10.1021/bi992781q 10820023

[bb16] Calvey, G. D., Katz, A. M., Schaffer, C. B. & Pollack, L. (2016). *Struct. Dyn.* **3**, 054301. 10.1063/1.4961971 PMC5010557 27679802

[bb17] Cepus, V., Ulbrich, C., Allin, C., Troullier, A. & Gerwert, K. (1998). *Methods Enzymol.* **291**, 223–245. 10.1016/s0076-6879(98)91015-1 9661152

[bb18] Chapman, H. N., Fromme, P., Barty, A., White, T. A., Kirian, R. A., Aquila, A., Hunter, M. S., Schulz, J., DePonte, D. P., Weierstall, U., Doak, R. B., Maia, F. R. N. C., Martin, A. V., Schlichting, I., Lomb, L., Coppola, N., Shoeman, R. L., Epp, S. W., Hartmann, R., Rolles, D., Rudenko, A., Foucar, L., Kimmel, N., Weidenspointner, G., Holl, P., Liang, M., Barthelmess, M., Caleman, C., Boutet, S., Bogan, M. J., Krzywinski, J., Bostedt, C., Bajt, S., Gumprecht, L., Rudek, B., Erk, B., Schmidt, C., Hömke, A., Reich, C., Pietschner, D., Strüder, L., Hauser, G., Gorke, H., Ullrich, J., Herrmann, S., Schaller, G., Schopper, F., Soltau, H., Kühnel, K.-U., Messer­schmidt, M., Bozek, J. D., Hau-Riege, S. P., Frank, M., Hampton, C. Y., Sierra, R. G., Starodub, D., Williams, G. J., Hajdu, J., Tîmneanu, N., Seibert, M. M., Andreasson, J., Rocker, A., Jönsson, O., Svenda, M., Stern, S., Nass, K., Andritschke, R., Schröter, C.-D., Krasniqi, F., Bott, M., Schmidt, K. E., Wang, X., Grotjohann, I., Holton, J. M., Barends, T. R. M., Neutze, R., Marchesini, S., Fromme, R., Schorb, S., Rupp, D., Adolph, M., Gorkhover, T., Andersson, I., Hirsemann, H., Potdevin, G., Graafsma, H., Nilsson, B. & Spence, J. C. H. (2011). *Nature*, **470**, 73–77.

[bb19] Chaves, O. A., Jesus, C. S. H., Cruz, P. F., Sant’Anna, C. M. R., Brito, R. M. M. & Serpa, C. (2016). *Spectrochim. Acta A Mol. Biomol. Spectrosc.* **169**, 175–181. 10.1016/j.saa.2016.06.028 27376757

[bb20] Cheng, R. K. (2020). *Crystals*, **10**, 215.

[bb21] Cho, H. S., Schotte, F., Dashdorj, N., Kyndt, J., Henning, R. & Anfinrud, P. A. (2016). *J. Am. Chem. Soc.* **138**, 8815–8823. 10.1021/jacs.6b03565 PMC5336379 27305463

[bb22] Claesson, E., Wahlgren, W. Y., Takala, H., Pandey, S., Castillon, L., Kuznetsova, V., Henry, L., Panman, M., Carrillo, M., Kübel, J., Nanekar, R., Isaksson, L., Nimmrich, A., Cellini, A., Morozov, D., Maj, M., Kurttila, M., Bosman, R., Nango, E., Tanaka, R., Tanaka, T., Fangjia, L., Iwata, S., Owada, S., Moffat, K., Groenhof, G., Stojković, E. A., Ihalainen, J. A., Schmidt, M. & Westenhoff, S. (2020). *eLife*, **9**, e53514.

[bb23] Coquelle, N., Sliwa, M., Woodhouse, J., Schirò, G., Adam, V., Aquila, A., Barends, T. R. M., Boutet, S., Byrdin, M., Carbajo, S., De la Mora, E., Doak, R. B., Feliks, M., Fieschi, F., Foucar, L., Guillon, V., Hilpert, M., Hunter, M. S., Jakobs, S., Koglin, J. E., Kovácsová, G., Lane, T. J., Lévy, B., Liang, M., Nass, K., Ridard, J., Robinson, J. S., Roome, C. M., Ruckebusch, C., Seaberg, M., Thepaut, M., Cammarata, M., Demachy, I., Field, M., Shoeman, R. L., Bourgeois, D., Colletier, J.-P., Schlichting, I. & Weik, M. (2018). *Nat. Chem.* **10**, 31–37. 10.1038/nchem.2853 29256511

[bb24] Corrie, J. E. T., Furuta, T., Givens, R., Yousef, A. L. & Goeldner, M. (2005). *Dynamic Studies in Biology: Phototriggers, Photoswitches and Caged Biomolecules*, edited by M. Goeldner & R. Givens, pp. 1–94. Weinheim: Wiley-VCH.

[bb25] Deisenhofer, J. & Michel, H. (1989). *Science*, **245**, 1463–1473. 10.1126/science.245.4925.1463 17776797

[bb26] Dixit, N. S. & Mackay, R. A. (1983). *J. Am. Chem. Soc.* **105**, 2928–2929.

[bb27] Ellis-Davies, G. C., Kaplan, J. H. & Barsotti, R. J. (1996). *Biophys. J.* **70**, 1006–1016. 10.1016/S0006-3495(96)79644-3 PMC1225001 8789118

[bb28] Filevich, O., Salierno, M. & Etchenique, R. (2010). *J. Inorg. Biochem.* **104**, 1248–1251. 10.1016/j.jinorgbio.2010.08.003 20825994

[bb29] Fromme, P. (2015). *Nat. Chem. Biol.* **11**, 895–899. 10.1038/nchembio.1968 PMC4839532 26575227

[bb30] Furuta, T. & Iwamura, M. (1998). *Methods Enzymol.* **291**, 50–63. 10.1016/s0076-6879(98)91006-0 9661144

[bb31] Furuta, T., Wang, S. S.-H., Dantzker, J. L., Dore, T. M., Bybee, W. J., Callaway, E. M., Denk, W. & Tsien, R. Y. (1999). *Proc. Natl Acad. Sci. USA*, **96**, 1193–1200. 10.1073/pnas.96.4.1193 PMC15439 9990000

[bb32] Geissler, D., Kresse, W., Wiesner, B., Bendig, J., Kettenmann, H. & Hagen, V. (2003). *ChemBioChem*, **4**, 162–170. 10.1002/cbic.200390027 12616629

[bb33] Goeldner, M. & Givens, R. (2006). Editors. *Dynamic Studies in Biology: Phototriggers, Photoswitches and Caged Biomolecules*. Weinheim: Wiley-VCH.

[bb34] Gorel, A., Schlichting, I. & Barends, T. R. M. (2021). *IUCrJ*, **8**, 532–543. 10.1107/S205225252100467X PMC8256713 34258002

[bb150] Grewer, C., Jäger, J., Carpenter, B. K. & Hess, G. P. (2000). *Biochemistry*, **39**, 2063–2070. 10.1021/bi9919652 10684656

[bb35] Grünbein, M. L. & Nass Kovacs, G. (2019). *Acta Cryst.* D**75**, 178–191. 10.1107/S205979831801567X PMC6400261 30821706

[bb36] Grünbein, M. L., Stricker, M., Nass Kovacs, G., Kloos, M., Doak, R. B., Shoeman, R. L., Reinstein, J., Lecler, S., Haacke, S. & Schlichting, I. (2020). *Nat. Methods*, **17**, 681–684. 10.1038/s41592-020-0847-3 32451477

[bb37] Hagen, V., Bendig, J., Frings, S., Eckardt, T., Helm, S., Reuter, D. & Kaupp, U. B. (2001). *Angew. Chem. Int. Ed.* **40**, 1045–1048. 10.1002/1521-3773(20010316)40:6<1045::aid-anie10450>3.0.co;2-f 11268067

[bb38] Hagen, V., Benndorf, K., Kaupp, U. B., Pavlos, C. M., Xu, H., Toscano, J. P., Hess, G. P., Gillespie, D. C., Kim, G. & Kandler, K. (2005). *Dynamic Studies in Biology: Phototriggers, Photoswitches and Caged Biomolecules*, edited by M. Goeldman & R. Givens, pp. 155–251. Weinheim: Wiley-VCH.

[bb39] Hagen, V., Dekowski, B., Nache, V., Schmidt, R., Geissler, D., Lorenz, D., Eichhorst, J., Keller, S., Kaneko, H., Benndorf, K. & Wiesner, B. (2005). *Angew. Chem. Int. Ed.* **44**, 7887–7891. 10.1002/anie.200502411 16270373

[bb40] Hajdu, J., Machin, P. A., Campbell, J. W., Greenhough, T. J., Clifton, I. J., Zurek, S., Gover, S., Johnson, L. N. & Elder, M. (1987). *Nature*, **329**, 178–181. 10.1038/329178a0 3114644

[bb41] Hauke, S., Dutta, A. K., Eisenbeis, V. B., Bezold, D., Bittner, T., Wittwer, C., Thakor, D., Pavlovic, I., Schultz, C. & Jessen, H. J. (2019). *Chem. Sci.* **10**, 2687–2692. 10.1039/c8sc03479f PMC6419925 30996985

[bb42] Havrylyuk, D., Stevens, K., Parkin, S. & Glazer, E. C. (2020). *Inorg. Chem.* **59**, 1006–1013. 10.1021/acs.inorgchem.9b02065 PMC8607748 31899619

[bb43] Helliwell, J. R. & Rentzepis, P. M. (1997). *Time-Resolved Diffraction.* Oxford: Clarendon Press.

[bb44] Heyes, D. J., Hardman, S. J. O., Pedersen, M. N., Woodhouse, J., De La Mora, E., Wulff, M., Weik, M., Cammarata, M., Scrutton, N. S. & Schirò, G. (2019). *Commun. Biol.* **2**, 1. 10.1038/s42003-018-0242-0 PMC6318211 30740537

[bb45] Howard-Jones, A. R., Adam, V., Cowley, A., Baldwin, J. E. & Bourgeois, D. (2009). *Photochem. Photobiol. Sci.* **8**, 1150–1156. 10.1039/b821516b 19639117

[bb46] Josts, I., Niebling, S., Gao, Y., Levantino, M., Tidow, H. & Monteiro, D. (2018). *IUCrJ*, **5**, 667–672. 10.1107/S2052252518012149 PMC6211537 30443351

[bb47] Kaplan, J. H. & Ellis-Davies, G. C. (1988). *Proc. Natl Acad. Sci. USA*, **85**, 6571–6575. 10.1073/pnas.85.17.6571 PMC282015 3137570

[bb48] Kaplan, J. H., Forbush, B. & Hoffman, J. F. (1978). *Biochemistry*, **17**, 1929–1935. 10.1021/bi00603a020 148906

[bb49] Klán, P., Šolomek, T., Bochet, C. G., Blanc, A., Givens, R., Rubina, M., Popik, V., Kostikov, A. & Wirz, J. (2013). *Chem. Rev.* **113**, 119–191. 10.1021/cr300177k PMC3557858 23256727

[bb50] Kohse, S., Neubauer, A., Pazidis, A., Lochbrunner, S. & Kragl, U. (2013). *J. Am. Chem. Soc.* **135**, 9407–9411. 10.1021/ja400700x 23688056

[bb51] Kramer, R. H., Chambers, J. J. & Trauner, D. (2005). *Nat. Chem. Biol.* **1**, 360–365. 10.1038/nchembio750 16370371

[bb52] Kubelka, J. (2009). *Photochem. Photobiol. Sci.* **8**, 499. 10.1039/b819929a 19337664

[bb53] Kumar, G. S. & Neckers, D. C. (1989). *Chem. Rev.* **89**, 1915–1925.

[bb54] Kupitz, C., Basu, S., Grotjohann, I., Fromme, R., Zatsepin, N. A., Rendek, K. N., Hunter, M. S., Shoeman, R. L., White, T. A., Wang, D., James, D., Yang, J.-H., Cobb, D. E., Reeder, B., Sierra, R. G., Liu, H., Barty, A., Aquila, A. L., Deponte, D., Kirian, R. A., Bari, S., Bergkamp, J. J., Beyerlein, K. R., Bogan, M. J., Caleman, C., Chao, T.-C., Conrad, C. E., Davis, K. M., Fleckenstein, H., Galli, L., Hau-Riege, S. P., Kassemeyer, S., Laksmono, H., Liang, M., Lomb, L., Marchesini, S., Martin, A. V., Messerschmidt, M., Milathianaki, D., Nass, K., Ros, A., Roy-Chowdhury, S., Schmidt, K., Seibert, M., Steinbrener, J., Stellato, F., Yan, L., Yoon, C., Moore, T. A., Moore, A. L., Pushkar, Y., Williams, G. J., Boutet, S., Doak, R. B., Weierstall, U., Frank, M., Chapman, H. N., Spence, J. C. H. & Fromme, P. (2014). *Nature*, **513**, 261–265.

[bb55] Levantino, M., Schirò, G., Lemke, H. T., Cottone, G., Glownia, J. M., Zhu, D., Chollet, M., Ihee, H., Cupane, A. & Cammarata, M. (2015). *Nat. Commun.* **6**, 6772. 10.1038/ncomms7772 PMC4396393 25832715

[bb56] Levantino, M., Yorke, B. A., Monteiro, D. C. F., Cammarata, M. & Pearson, A. R. (2015). *Curr. Opin. Struct. Biol.* **35**, 41–48. 10.1016/j.sbi.2015.07.017 26342489

[bb57] Lewis, F. D., Wu, Y. & Liu, X. (2002). *J. Am. Chem. Soc.* **124**, 12165–12173. 10.1021/ja026941o 12371856

[bb58] Li, A., Turro, C. & Kodanko, J. J. (2018). *Chem. Commun.* **54**, 1280–1290. 10.1039/c7cc09000e PMC5904840 29323683

[bb59] Liao, Y. (2017). *Acc. Chem. Res.* **50**, 1956–1964. 10.1021/acs.accounts.7b00190 28692282

[bb60] Ludovici, C., Fröhlich, R., Vogtt, K., Mamat, B. & Lübben, M. (2002). *Eur. J. Biochem.* **269**, 2630–2637. 10.1046/j.1432-1033.2002.02944.x 12027903

[bb61] Mahmoodi, M. M., Abate-Pella, D., Pundsack, T. J., Palsuledesai, C. C., Goff, P. C., Blank, D. A. & Distefano, M. D. (2016). *J. Am. Chem. Soc.* **138**, 5848–5859. 10.1021/jacs.5b11759 PMC5026405 27027927

[bb62] Makinen, M. W. & Fink, A. L. (1977). *Annu. Rev. Biophys. Bioeng.* **6**, 301–343. 10.1146/annurev.bb.06.060177.001505 194529

[bb63] Malmerberg, E., Bovee-Geurts, P. H. M., Katona, G., Deupi, X., Arnlund, D., Wickstrand, C., Johansson, L. C., Westenhoff, S., Nazarenko, E., Schertler, G. F. X., Menzel, A., de Grip, W. J. & Neutze, R. (2015). *Sci. Signal.* **8**, ra26. 10.1126/scisignal.2005646 25759477

[bb64] Martiel, I., Müller-Werkmeister, H. M. & Cohen, A. E. (2019). *Acta Cryst.* D**75**, 160–177. 10.1107/S2059798318017953 PMC6400256 30821705

[bb65] Martin-Garcia, J. M., Conrad, C. E., Nelson, G., Stander, N., Zatsepin, N. A., Zook, J., Zhu, L., Geiger, J., Chun, E., Kissick, D., Hilgart, M. C., Ogata, C., Ishchenko, A., Nagaratnam, N., Roy-Chowdhury, S., Coe, J., Subramanian, G., Schaffer, A., James, D., Ketwala, G., Venugopalan, N., Xu, S., Corcoran, S., Ferguson, D., Weierstall, U., Spence, J. C. H., Cherezov, V., Fromme, P., Fischetti, R. F. & Liu, W. (2017). *IUCrJ*, **4**, 439–454. 10.1107/S205225251700570X PMC5571807 28875031

[bb66] Mayer, G. & Heckel, A. (2006). *Angew. Chem. Int. Ed.* **45**, 4900–4921. 10.1002/anie.200600387 16826610

[bb67] Mehrabi, P., Schulz, E. C., Dsouza, R., Müller-Werkmeister, H. M., Tellkamp, F., Miller, R. J. D. & Pai, E. F. (2019). *Science*, **365**, 1167–1170. 10.1126/science.aaw9904 31515393

[bb68] Moffat, K. (1998). *Nat. Struct. Mol. Biol.* **5**, 641–643. 10.1038/1333 9699613

[bb69] Moffat, K. (2001). *Chem. Rev.* **101**, 1569–1582. 10.1021/cr990039q 11709992

[bb70] Moffat, K. (2019). *Phil. Trans. R. Soc. A*, **377**, 20180243. 10.1098/rsta.2018.0243 PMC6501890 31030647

[bb71] Momotake, A., Lindegger, N., Niggli, E., Barsotti, R. J. & Ellis-Davies, G. C. R. (2006). *Nat. Methods*, **3**, 35–40. 10.1038/nmeth821 16369551

[bb72] Monroe, W. T., McQuain, M. M., Chang, M. S., Alexander, J. S. & Haselton, F. R. (1999). *J. Biol. Chem.* **274**, 20895–20900. 10.1074/jbc.274.30.20895 10409633

[bb73] Monteiro, D. C. F., Vakili, M., Harich, J., Sztucki, M., Meier, S. M., Horrell, S., Josts, I. & Trebbin, M. (2019). *J. Synchrotron Rad.* **26**, 406–412. 10.1107/S1600577519000304 30855249

[bb74] Monteiro, D. C. F., von Stetten, D., Stohrer, C., Sans, M., Pearson, A. R., Santoni, G., van der Linden, P. & Trebbin, M. (2020). *IUCrJ*, **7**, 207–219. 10.1107/S2052252519016865 PMC7055382 32148849

[bb75] Namiki, S., Arai, T. & Fujimori, K. (1997). *J. Am. Chem. Soc.* **119**, 3840–3841.

[bb76] Nandi, R., Yucknovsky, A., Mazo, M. M. & Amdursky, N. (2020). *J. Mater. Chem. B*, **8**, 6964–6974. 10.1039/d0tb00818d 32500877

[bb77] Neutze, R. (2014). *Philos. Trans. R. Soc. B*, **369**, 20130318. 10.1098/rstb.2013.0318 PMC4052859 24914150

[bb78] Neutze, R. & Moffat, K. (2012). *Curr. Opin. Struct. Biol.* **22**, 651–659. 10.1016/j.sbi.2012.08.006 PMC3520507 23021004

[bb79] Nogly, P., Weinert, T., James, D., Carbajo, S., Ozerov, D., Furrer, A., Gashi, D., Borin, V., Skopintsev, P., Jaeger, K., Nass, K., Båth, P., Bosman, R., Koglin, J., Seaberg, M., Lane, T., Kekilli, D., Brünle, S., Tanaka, T., Wu, W., Milne, C., White, T., Barty, A., Weierstall, U., Panneels, V., Nango, E., Iwata, S., Hunter, M., Schapiro, I., Schertler, G., Neutze, R. & Standfuss, J. (2018). *Science*, **361**, eaat0094. 10.1126/science.aat0094 29903883

[bb80] Nomura, T., Kimura, T., Kanematsu, Y., Yamada, D., Yamashita, K., Hirata, K., Ueno, G., Murakami, H., Hisano, T., Yamagiwa, R., Takeda, H., Gopalasingam, C., Kousaka, R., Yanagisawa, S., Shoji, O., Kumasaka, T., Yamamoto, M., Takano, Y., Sugimoto, H., Tosha, T., Kubo, M. & Shiro, Y. (2021). *Proc. Natl Acad. Sci. USA*, **118**, e2101481118. 10.1073/pnas.2101481118 PMC8166195 34001620

[bb81] Pande, K., Hutchison, C. D. M., Groenhof, G., Aquila, A., Robinson, J. S., Tenboer, J., Basu, S., Boutet, S., DePonte, D. P., Liang, M., White, T. A., Zatsepin, N. A., Yefanov, O., Morozov, D., Oberthuer, D., Gati, C., Subramanian, G., James, D., Zhao, Y., Koralek, J., Brayshaw, J., Kupitz, C., Conrad, C., Roy-Chowdhury, S., Coe, J. D., Metz, M., Xavier, P. L., Grant, T. D., Koglin, J. E., Ketawala, G., Fromme, R., Šrajer, V., Henning, R., Spence, J. C. H., Ourmazd, A., Schwander, P., Weierstall, U., Frank, M., Fromme, P., Barty, A., Chapman, H. N., Moffat, K., van Thor, J. J. & Schmidt, M. (2016). *Science*, **352**, 725–729.

[bb82] Pavlovic, I., Thakor, D. T., Vargas, J. R., McKinlay, C. J., Hauke, S., Anstaett, P., Camuña, R. C., Bigler, L., Gasser, G., Schultz, C., Wender, P. A. & Jessen, H. J. (2016). *Nat. Commun.* **7**, 10622. 10.1038/ncomms10622 PMC4743007 26842801

[bb83] Renner, C. & Moroder, L. (2006). *ChemBioChem*, **7**, 868–878. 10.1002/cbic.200500531 16642526

[bb84] Roedig, P., Duman, R., Sanchez-Weatherby, J., Vartiainen, I., Burkhardt, A., Warmer, M., David, C., Wagner, A. & Meents, A. (2016). *J. Appl. Cryst.* **49**, 968–975. 10.1107/S1600576716006348 PMC4886986 27275143

[bb85] Roy, S., Patra, A. K., Dhar, S. & Chakravarty, A. R. (2008). *Inorg. Chem.* **47**, 5625–5633. 10.1021/ic702508r 18533626

[bb86] Schatzschneider, U. (2010). *Eur. J. Inorg. Chem.* **2010**, 1451–1467.

[bb87] Schmidt, M. (2013). *Adv. Condens. Matter Phys.* **2013**, 1–10.

[bb88] Schmidt, M. (2017). *Struct. Dyn.* **4**, 032201. 10.1063/1.4974172 PMC5291790 28191482

[bb89] Schotte, F., Cho, H. S., Kaila, V. R. I., Kamikubo, H., Dashdorj, N., Henry, E. R., Graber, T. J., Henning, R., Wulff, M., Hummer, G., Kataoka, M. & Anfinrud, P. A. (2012). *Proc. Natl Acad. Sci. USA*, **109**, 19256–19261. 10.1073/pnas.1210938109 PMC3511082 23132943

[bb90] Schotte, F., Lim, M., Jackson, T. A., Smirnov, A. V., Soman, J., Olson, J. S. Jr, Phillips, G. N., Wulff, M. & Anfinrud, P. A. (2003). *Science*, **300**, 1944–1947. 10.1126/science.1078797 12817148

[bb91] Schulz, E. C., Mehrabi, P., Müller-Werkmeister, H. M., Tellkamp, F., Jha, A., Stuart, W., Persch, E., De Gasparo, R., Diederich, F., Pai, E. F. & Miller, R. J. D. (2018). *Nat. Methods*, **15**, 901–904. 10.1038/s41592-018-0180-2 30377366

[bb92] Šrajer, V. & Royer, W. E. (2008). *Methods Enzymol.* **437**, 379–395. 10.1016/S0076-6879(07)37019-5 PMC3287071 18433638

[bb93] Šrajer, V. & Schmidt, M. (2017). *J. Phys. D Appl. Phys.* **50**, 373001. 10.1088/1361-6463/aa7d32 PMC5771432 29353938

[bb94] Sui, S. & Perry, S. L. (2017). *Struct. Dyn.* **4**, 032202.

[bb95] Szymański, W., Beierle, J. M., Kistemaker, H. A. V., Velema, W. A. & Feringa, B. L. (2013). *Chem. Rev.* **113**, 6114–6178. 10.1021/cr300179f 23614556

[bb96] Thompson, M. C., Barad, B. A., Wolff, A. M., Cho, H. S., Schotte, F., Schwarz, D. M. C., Anfinrud, P. & Fraser, J. S. (2019). *Nat. Chem.* **11**, 1058–1066. 10.1038/s41557-019-0329-3 PMC6815256 31527847

[bb97] Tosha, T., Nomura, T., Nishida, T., Saeki, N., Okubayashi, K., Yamagiwa, R., Sugahara, M., Nakane, T., Yamashita, K., Hirata, K., Ueno, G., Kimura, T., Hisano, T., Muramoto, K., Sawai, H., Takeda, H., Mizohata, E., Yamashita, A., Kanematsu, Y., Takano, Y., Nango, E., Tanaka, R., Nureki, O., Shoji, O., Ikemoto, Y., Murakami, H., Owada, S., Tono, K., Yabashi, M., Yamamoto, M., Ago, H., Iwata, S., Sugimoto, H., Shiro, Y. & Kubo, M. (2017). *Nat. Commun.* **8**, 1585. 10.1038/s41467-017-01702-1 PMC5691058 29147002

[bb98] Volgraf, M., Gorostiza, P., Numano, R., Kramer, R. H., Isacoff, E. Y. & Trauner, D. (2006). *Nat. Chem. Biol.* **2**, 47–52. 10.1038/nchembio756 PMC1447676 16408092

[bb99] Waldeck, D. H. (1991). *Chem. Rev.* **91**, 415–436.

[bb100] Wieboldt, R., Gee, K. R., Niu, L., Ramesh, D., Carpenter, B. K. & Hess, G. P. (1994). *Proc. Natl Acad. Sci. USA*, **91**, 8752–8756. 10.1073/pnas.91.19.8752 PMC44684 8090718

[bb101] Wöhri, A. B., Katona, G., Johansson, L. C., Fritz, E., Malmerberg, E., Andersson, M., Vincent, J., Eklund, M., Cammarata, M., Wulff, M., Davidsson, J., Groenhof, G. & Neutze, R. (2010). *Science*, **328**, 630–633. 10.1126/science.1186159 20431017

[bb102] Woodhouse, J., Nass Kovacs, G., Coquelle, N., Uriarte, L. M., Adam, V., Barends, T. R. M., Byrdin, M., de la Mora, E., Doak, R. B., Feliks, M., Field, M., Fieschi, F., Guillon, V., Jakobs, S., Joti, Y., Macheboeuf, P., Motomura, K., Nass, K., Owada, S., Roome, C. M., Ruckebusch, C., Schirò, G., Shoeman, R. L., Thepaut, M., Togashi, T., Tono, K., Yabashi, M., Cammarata, M., Foucar, L., Bourgeois, D., Sliwa, M., Colletier, J.-P., Schlichting, I. & Weik, M. (2020). *Nat. Commun.* **11**, 741. 10.1038/s41467-020-14537-0 PMC7005145 32029745

[bb103] Yoder, N., Jalali-Yazdi, F., Noreng, S., Houser, A., Baconguis, I. & Gouaux, E. (2020). *J. Struct. Biol.* **212**, 107624. 10.1016/j.jsb.2020.107624 PMC7959588 32950604

[bb104] Zaitsev-Doyle, J. J., Puchert, A., Pfeifer, Y., Yan, H., Yorke, B. A., Müller-Werkmeister, H. M., Uetrecht, C., Rehbein, J., Huse, N., Pearson, A. R. & Sans, M. (2019). *RSC Adv.* **9**, 8695–8699. 10.1039/c9ra00968j PMC9061760 35518684

[bb105] Zhu, M. & Zhou, H. (2018). *Org. Biomol. Chem.* **16**, 8434–8445. 10.1039/c8ob02157k 30375620

